# Discovery of novel conjugates of quinoline and thiazolidinone urea as potential anti-colorectal cancer agent

**DOI:** 10.1080/14756366.2022.2117318

**Published:** 2022-08-31

**Authors:** Li Xiong, Huan He, Mengmeng Fan, Liping Hu, Fei Wang, Xiaomeng Song, Shengmin Shi, Baohui Qi

**Affiliations:** a Department of Bioengineering, Zhuhai Campus of Zunyi Medical University, Zhuhai, China; b Key Laboratory of Biocatalysis & Chiral Drug Synthesis of Guizhou Province, Zunyi Medical University, Zunyi, China

**Keywords:** Anticancer, HGFR, MST1R, quinoline, structure modification

## Abstract

Based on the obtained SARs, further structural optimisation of compound BC2021-104511-15i was conducted in this investigation, and totally ten novel quinoline derivates were designed, synthesised and optimised for biological activity. Among them, compound **10a** displayed significant *in vitro* anticancer activity against COLO 205 cells with an IC_50_ value of 0.11 μM which was over 90-fold more potent than that of Regorafenib (IC_50_>10.0 μM) and Fruquintinib (IC_50_>10.0 μM). Furthermore, compound **10a** exhibited over 90-fold selectivity towards COLO 205 relative to human normal colorectal mucosa epithelial cell FHC cells. Flow cytometry study demonstrated that compound **10a** could induce apoptosis in COLO 205 cells, however, it could not induce cell cycle arrest in COLO 205 cells. The results of preliminary kinase profile study showed that compound **10a** was a potential HGFR and MST1R dual inhibitor, with IC_50_ values of 0.11 μM and 0.045 μM, respectively.

## 1. Introduction

Colorectal cancer (CRC) is one of the most predominant malignancies with a high mortality rate globally^1^. It is estimated that the number of CRC patients will reach 2.5 million in 2035^2^. Approximately 25% of CRC patients presented metastatic disease at diagnosis, while almost 50% of them will develop metastases. According to the statistics, the 5-year survival rate ranged from 90% to 14% if CRC is diagnosed at a localised or metastatic stage^3^.

**Figure 1.**
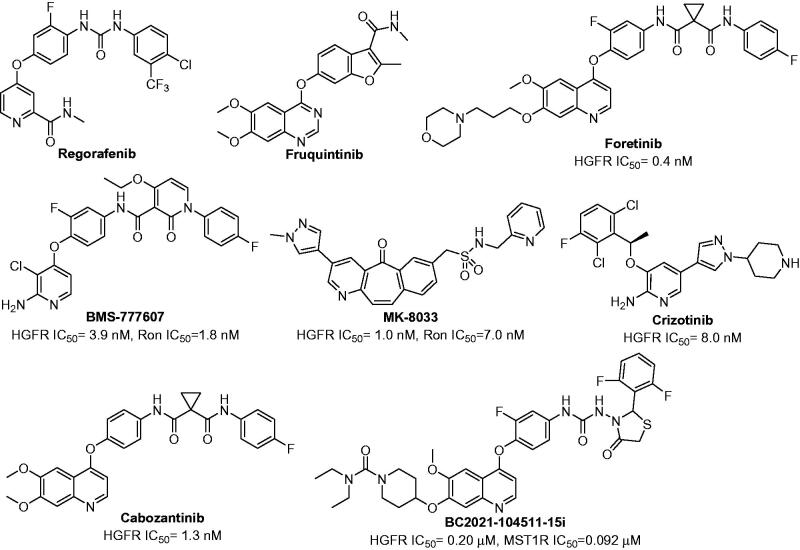
The structures of Regorafenib, Fruquintinib, Foretinib, BMS-777607, MK-8033, Crizotinib, Cabozantinib and BC2021-104511-15i.

Nowadays, chemotherapy is the most extensively applied approach for the treatment of primary CRC and/or metastatic CRC (mCRC)^4^. The drugs used in chemotherapy were divided into cytotoxic drugs, tyrosine kinase inhibitors (TKIs), monoclonal antibodies, and programmed cell death protein-1/programmed cell death 1 ligand 1 (PD1/PD-L1) inhibitors, *etc*^5^^,^^6^. Among them, TKIs could significantly improve major efficacy parameters which included response rate (RR), progression free survival (PFS) and overall survival (OS). Unfortunately, only Regorafenib and Fruquintinib are successfully utilised in clinic as TKIs for the treatment of patients with mCRC (Figure 1). Regorafenib is an orally bioavailable multitarget TKI which mainly inhibits the activity of vascular endothelial growth factor receptor1-3 (VEGFR1-3), tunica intima endothelial kinase 2 (TIE2), rearranged during transfection (RET), mast/stem cell growth factor receptor (Kit), and platelet-derived growth factor receptor (PDGFR), *etc*^7^. It has been approved by US Food and Drug Administration (US FDA) and European Medicines Agency (EMA) for the treatment of mCRC patients who had already been treated with fluoropyrimidine, oxaliplatin, anti-VEGF therapy and/or irinotecan-based chemotherapy^8^. Fruquintinib also is a small molecule multitarget TKI with high affinity for VEGFR1-3^9^. In 2018, it was approved by National Medical Products Administration (NMPA) for the treatment of mCRC patients who had suffered at least two unsuccessful standard therapies^10^. Despite the recent advances in the chemotherapy of primary CRC and mCRC, the survival benefit is still limited due to the high heterogeneity, resistance and severe side effects. Accordingly, there is still urgent need to develop alternative and potential therapeutic strategies with high efficacy and acceptable side effects for the treatment of CRC.

Hepatocyte growth factor receptor (HGFR, also known as mesenchymal-epithelial transition factor [c-Met]), and macrophage-stimulating protein receptor (MST1R, also known as recepteur d’Origine nantais [Ron]), belong to a unique subfamily of receptor tyrosine kinases (RTKs)^11^. It was reported that they shared 34% overall homology and the tyrosine kinase region shared 80% homology^12^. The phosphorylated HGFR and MST1R could activate several transduction proteins and trigger their downstream signalling cascades which mainly included phosphatidylin-ositol-3-kinase/protein kinase B/mammalian target of rapamycin (PI3K/AKT/mTOR) pathway, mitogen-activated protein kinase (MAPK) pathway, signal transducer and activator of transcription (STAT) pathway, cell sarcoma (c-Src), and extracellular regulated protein kinase1/2 (ERK1/2), *etc*^13–17^. The dysregulation of HGFR and/or MST1R were extensively implicated in multiple cancer oncogenic processes, such as proliferation, migration, invasion, angiogenesis, epithelial-to-mesenchymal transition (EMT) and drug resistance, *etc*^18–22^. It is noteworthy that HGFR and MST1R played an important role in the CRC’s progression, malignancy, and stemness. Therefore, HGFR/MST1R dual inhibitors might be optimal agents for the treatment of CRC. Pharmaceutically, numerous HGFR and/or MST1R kinase inhibitors have been evaluated for the treatment of different types of cancers, such as Foretinib, BMS-777607, MK-8033, Crizotinib, Cabozantinib (Figure 1)^23–25^. However, no small molecular HGFR and MST1R dual inhibitors have been discovered as agents for the treatment of CRC.

**Figure 2.**
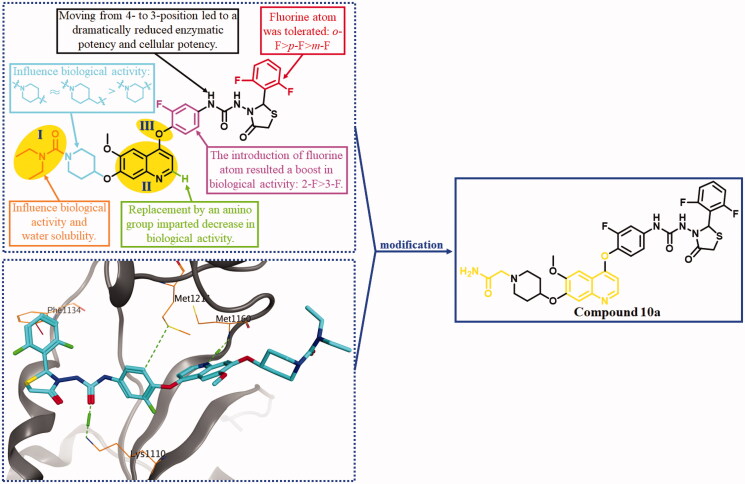
The binding mode of BC2021-104511-15i with HGFR (PBD ID: 3LQ8), the SARs of lead compound BC2021-104511-15i and the modification of fragments **I**, **II** and **III**. Lead compound was shown by blue sticks, and the H-bonds were represented by green dotted lines, and the H-arene interaction was shown by red dotted lines.

Based on the above survey, a study on developing novel HGFR/MST1R dual inhibitors as anti-CRC agents was carried out by our group. As shown in Figure 1, HGFR/MST1R dual inhibitor BC2021-104511-15i was discovered by our group^26^^,^^27^. It exhibited potential *in vitro* anticancer activity against several cancer cell lines, especially human colorectal carcinoma cell line HT-29 cells. In order to obtain a more potent HGFR/MST1R dual inhibitor as an agent for the treatment of CRC, further modification on the fragments **I**, **II**, and **III** of BC2021-104511-15i was performed (Figure 2). The details of design, synthesis, biological evaluation, docking study and anticancer mechanism were all discussed in the following sections.

## 2. The modification of lead compound

As shown in Figure 2, preliminary SARs were summarised based on the biological evaluation in our previous research. The SARs indicated that the groups attached to the piperidine ring (**I**) could significantly influence the HGFR and MST1R kinases inhibitory activity, *in vitro* anticancer activity and water solubility. Thus, compounds **10a–h** bearing variant substituents on piperidine rings were designed and synthesised in the beginning of this work. Docking study showed that two significant H-bonds were formed by quinoline ring and urea moiety with Met1160 and Lys1110, respectively (Figure 2). The terminal difluoro-substituted phenyl ring reached into a hydrophobic pocket, and H-π interaction was formed. We assumed that the spatial position change of the H-bond donors and acceptors in the moiety of thiazolidine-4-one urea might strengthen the H-bond and/or lead to additional H-bonds. Thus, the oxygen atom linked quinoline ring and 2-fluorophenyl ring (**III**) was replaced to deflect the dihedral angel formed by the aromatic rings, and compound **10i** was designed and synthesised. Additionally, a quinazoline derivate **19** was designed and prepared to investigate the influence on the kinase inhibitory activity when the electron density distribution changed in the quinoline ring.

Based on the above assumption, totally ten novel compounds were designed, synthesised and evaluated for their biological activity in the present work. Moreover, the anticancer mechanism was also investigated preliminarily.

## 3. Results and discussion

### 3.1. Chemistry

**Scheme 1.**
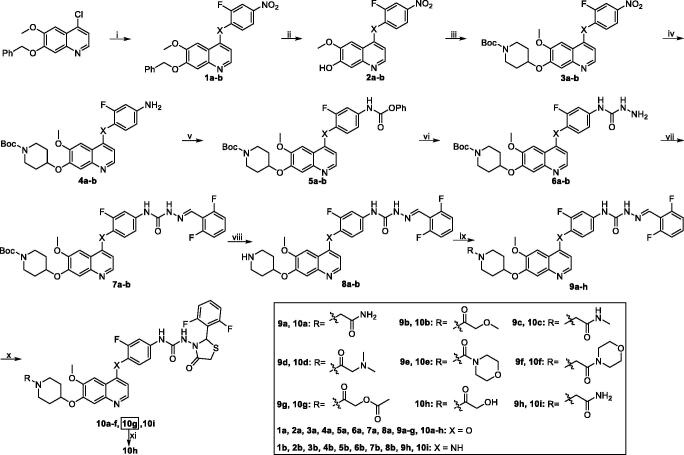
Synthesis of target compounds **10a–i**. Reagents and conditions: (i) **1a:** 2-fluoro-4-nitrophenol, PhCl, reflux, 14 h; **1b:** 2-fluoro-4-nitroaniline, *i*-PrOH, conc. HCl, reflux, 3 h; (ii) 33% HBr in HOAc, rt, 3 h; (iii) 1-Boc-4-methanesulfonyloxypiperidine, Cs_2_CO_3_, DMF, 110 °C, 6 h; (iv) Fe, 90% EtOH-H_2_O, conc. HCl (cat.), reflux, 4–6 h; (v) phenyl chloroformate, pyridine, CH_2_Cl_2_, rt, 2 h; (vi) 50% hydrazine hydrate, xylene, 70 °C, 2 h; (vii) 2, 6-difluorobenzaldehyde, *i*-PrOH, HOAc (cat.), reflux, 2–3 h; (viii) CF_3_COOH, CH_2_Cl_2_, rt, 2 h; (ix) RCOCl, Et_3_N, CH_2_Cl_2_, rt, 4–5 h; R-Cl, Cs_2_CO_3_, DMF, 90 °C, 6–8 h; (x) mercaptoacetic acid, SiCl_4_, CH_2_Cl_2_, reflux, 6–8 h; (xi) MeOH, NaOH, 50 °C, 1 h.

**Scheme 2.**
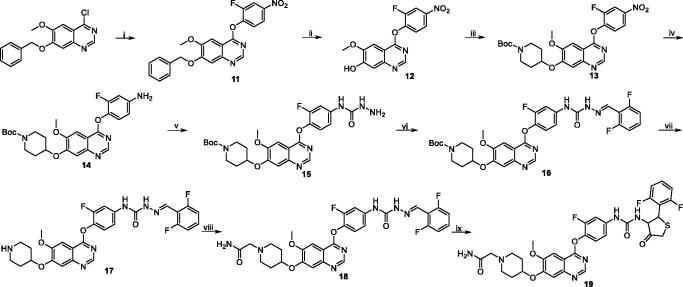
Synthesis of target compound **19**. Reagents and conditions: (i) 2-fluoro-4-nitrophenol, PhCl, reflux, 14 h; (ii) 33% HBr in HOAc, rt, 3 h; (iii) 1-Boc-4-methanesulfonyloxypiperidine, Cs_2_CO_3_, DMF, 110 °C, 6 h; (iv) Fe, 90% EtOH-H_2_O, conc. HCl (cat.), reflux, 4 h; (v) (1) phenyl chloroformate, pyridine, CH_2_Cl_2_, rt, 2 h; (2) 50% hydrazine hydrate, xylene, 70 °C, 2 h; (vi) 2, 6-difluorobenzaldehyde, *i*-PrOH, HOAc (cat.), reflux, 3 h; (vii) CF_3_COOH, CH_2_Cl_2_, rt, 2 h; (viii) 2-chloroacetamide, Cs_2_CO_3_, DMF, 90 °C, 8 h; (ix) mercaptoacetic acid, SiCl_4_, CH_2_Cl_2_, reflux, 6 h.

Target compounds **10a–i** and **19** were successfully prepared by the synthetic routes outlined in Scheme 1 and Scheme 2^26^. Commercially available 7-(benzyloxy)-4-chloro-6-methoxyquinoline was reacted with 2-fluoro-4-nitrophenol in refluxing chlorobenzene to afford 4-aryloxyquinoline **1a**. Subsequently, intermediate **1** was cleanly debenzylated by 33% HBr in acetic acid to obtain phenol **2a**, which was alkylated with 1-Boc-4-methanesulfonyloxypiperidine in the presence of Cs_2_CO_3_ to provide **3a**. The amine **4a** was achieved by reduction of nitro group in **3a**. Then, the semicarbazide **6a** was acquired using a two-step procedure involving acylation reaction with phenyl chloroformate in the presence of pyridine and subsequent hydrazinolysis reaction with 50% hydrazine hydrate in xylene with vigorous agitation. Condensation of **6a** with 2,6-difluorobenzaldehyde in favour of catalytic HOAc was carried out to provide **7a**. The *N*-Boc group in the semicarbazone **7a** was deprotected by CF_3_COOH to afford piperidine derivate **8a**, which was then acylated or alkylated with corresponding acyl chlorides or chlorides to give intermediates **9a–h**. Finally, the target compounds **10a–g** were prepared by cyclisation reaction with mercaptoacetic acid in the presence of SiCl_4_. Target compound **10h** was obtained by the hydrolysis of compound **10g** under basic condition in MeOH.

Reaction of commercially available 7-(benzyloxy)-4-chloro-6-methoxyquinoline with 2-fluoro-4-nitroaniline in favour of catalytic concentrated HCl gave 7-(benzyloxy)-*N*-(2-fluoro-4-nitrophenyl)-6-methoxyquinolin-4-amine **1b**^28^. In the following procedures, target compound **10i** was prepared by similar methods of the synthesis of compounds **10a–g**.

Taking commercially available 7-(benzyloxy)-4-chloro-6-methoxyquinazoline and 2-fluoro-4-nitrophenol as starting materials, quinazoline derivate **11** was acquired by nucleophilic substitution in refluxing chlorobenzene. Subsequently, target compound **19** was synthesised by similar routes outlined in Scheme 2.

### 3.2. Structure-Activity relationship

**Figure 3.**
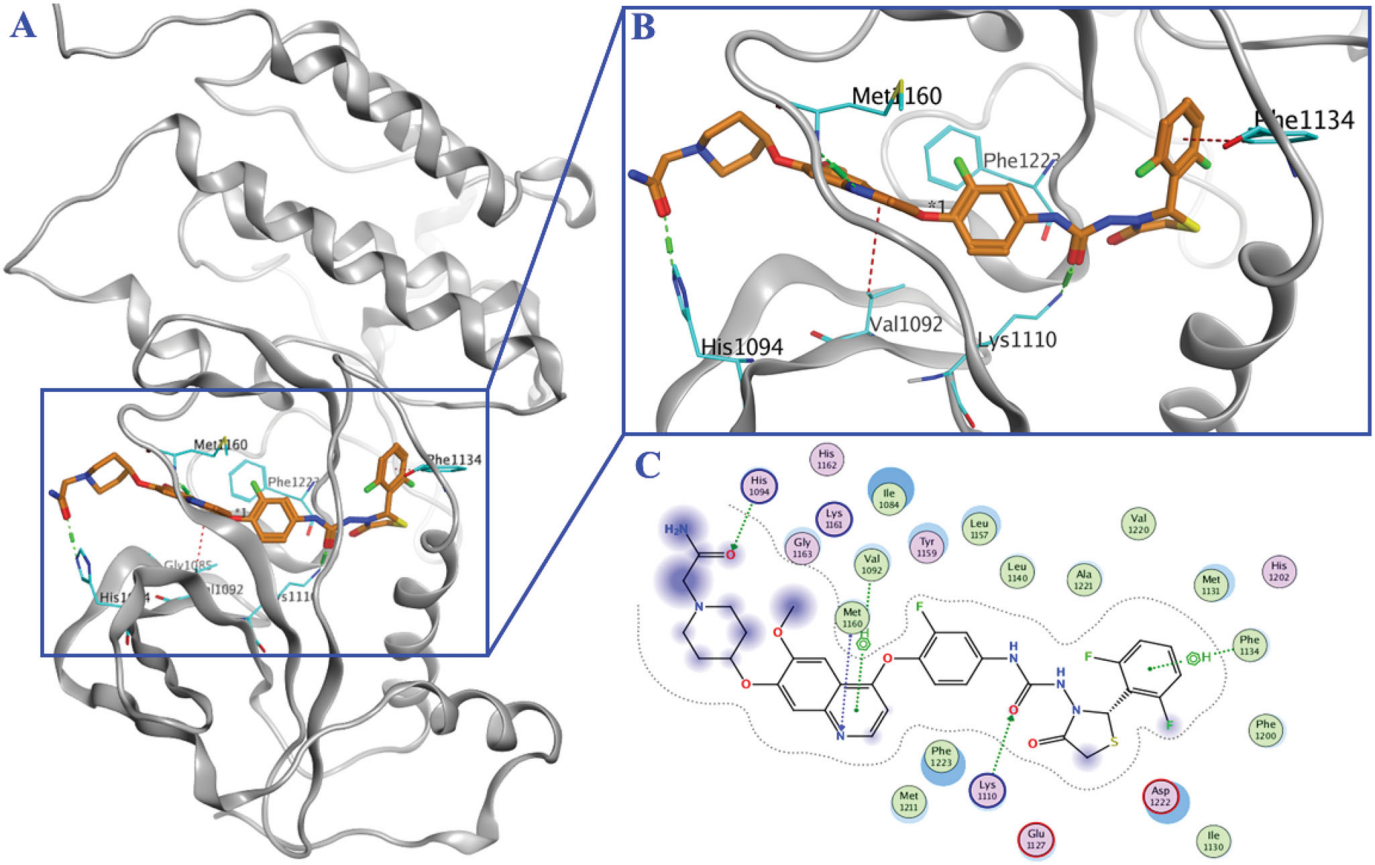
Mimetic binding mode of compound **10a** (yellow sticks) within HGFR (PDB ID: 3LQ8). The H-bonds were represented by green dotted line and the H-π was represented by red dotted lines.

Lead compound BC2021-104511-15i, Fruquintinib, Regorafenib, Cabozantinb and Foretinib were chosen as positive controls in the study of biological evaluation. In our previous research, it was revealed that the substituent on the piperidine ring could significantly influence the inhibitory activity against both kinases and cancer cells. Thus, eight novel compounds bearing diversified R groups (**10a–h**) were designed and synthesised. Biological activity study indicated that no obvious differences on kinase inhibitory activity could be found between the heterocyclic groups (**10e–f**) and catenoid groups (**10a–d** and **10g–h**). As a general trend, the introduction of N-CH_2_-CO fragment was beneficial for the HGFR and MST1R kinase inhibitory activity, such as compounds **10a**, **10c** and **10f**. Docking study showed that additional H-bond formed by the carbonyl group and the residue His1094 might lead to the increased activity (Figure 3). Among the eight compounds, compound **10a** was identified as the most potent HGFR and MST1R inhibitor, with IC_50_ values of 0.11 μM and 0.045 μM, respectively (Table 1).

**Table 1.** The structures of target compounds **10a–i** and **19** and their inhibitory activity against HGFR, MST1R, HT-29, HCT-116 and COLO 205 cells.

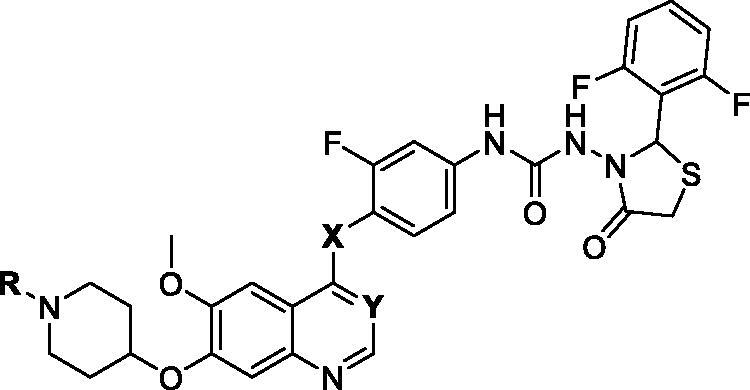								
Compd.	R	X	Y	IC50 (μM)				
				HGFR^a^	MST1R^a^	HT-29^b^	HCT-116^b^	COLO 205^b^
**10a**	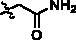	O	CH	0.11	0.045	1.3 ± 0.11	0.87 ± 0.11	0.11 ± 0.024
**10b**	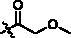	O	CH	0.18	0.085	3.0 ± 0.24	3.7 ± 0.34	0.31 ± 0.021
**10c**	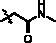	O	CH	0.14	0.072	4.1 ± 0.27	5.9 ± 0.53	0.44 ± 0.038
**10d**	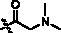	O	CH	0.90	0.44	2.9 ± 0.19	2.4 ± 0.26	4.9 ± 0.38
**10e**	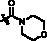	O	CH	0.61	0.15	7.7 ± 0.56	2.4 ± 0.26	0.48 ± 0.042
**10f**	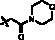	O	CH	0.14	0.078	1.9 ± 0.11	2.6 ± 0.27	0.42 ± 0.034
**10g**	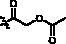	O	CH	0.18	0.11	6.9 ± 0.47	4.8 ± 0.44	0.59 ± 0.045
**10h**	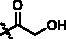	O	CH	0.22	0.16	6.5 ± 0.43	–	0.85 ± 0.073
**10i**	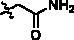	NH	CH	–	–	>10.0	>10.0	–
**19**	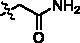	O	N	–	–	8.6 ± 0.67	–	5.3 ± 0.47
BC2021-104511-15i	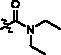	O	CH	0.27^c^	0.11^c^	0.79 ± 0.058^c^	4.8 ± 0.44	0.72 ± 0.065
Fruquintinib	–	–	–	–	–	1.57	2.2 ± 0.17	>10.0
Regorafenib	–	–	–	–	–	8.49	8.2 ± 0.75	>10.0
Cabozantinib	–	–	–	0.0019		–	–	–
Foretinib	–	–	–		0.0024	–	–	–

^a^The IC_50_ values are expressed as the mean of two independent experiments. ^b^The IC_50_ values were an average of three separate determinations and standard deviations were shown. ^c^Reported IC_50_ values were 0.20 μM (HGFR), 0.092 μM (MST1R) and 0.19 μM (HT-29 cells), respectively.

Compared with compound **10a**, replacement of the oxygen atom linked the quinoline ring and 2-fluorophenyl ring by NH (**10i**) led to a significant decrease in anticancer activity (HT-29 IC_50_>10.0 μM and HCT-116 IC_50_>10.0 μM). Additionally, the quinazoline derivate **19** also showed weaker anticancer activity (HT-29 IC_50_=8.6 μM and COLO 205 IC_50_=5.3 μM, Table 1). The decrease of the biological activity might result from the conversion of electron density distribution in the quinoline ring which might weaken the H-bond between the nitrogen atom in quinoline and the residue Met1160.

### 3.3. The cytotoxicity against FHC cells

In order to investigate the cell selectivity index, the cytotoxicity of potent compounds against human normal colorectal mucosa epithelial cell FHC cells was determined. As could be seen in Table 2, all the anticancer agents **10a–b**, **10d**, **10f** and **10h** displayed no obvious cytotoxicity against FHC cells (IC_50_>10.0 μM). Notably, the cell selectivity index of the most potent compound **10a** was over 90 (FHC IC_50_ value vs COLO 205 IC_50_ value).

**Table 2.** The cytotoxicity of selected compounds against FHC cells.

Cells	IC_50_ (μM)^a^				
	10a	10b	10d	10f	10h
FHC	>10.0	>10.0	>10.0	>10.0	>10.0

^a^The IC_50_ values were an average of three separate determinations.

### 3.4. Molecular docking study

Docking of the most potent compound **10a** into HGFR was performed by Molecular Operating Environment (MOE). As shown in Figure 3, compound **10a** adopted an extended conformation as type II kinase inhibitor exemplified by Cabozantinib and Foretinib. Totally three key hydrogen bonds were formed: nitrogen atom in quinoline with residue Met1160, oxygen atom in urea moiety with residue Lys1110, and oxygen atom in the terminal amido group with residue His1094. The hydrophobic pocket was occupied by the terminal 2,6-difluorophenyl ring, and weak H-π interaction was formed between 2,6-difluorophenyl fragment with residue Phe1134. Additionally, weak H-arene interaction was also formed between the quinoline ring and residue Val1092.

### 3.5. In vitro kinase profile

To further investigate the kinase selectivity of novel target compounds, the inhibitory activity of compounds **10a** and **10b** against another seven kinases was evaluated, including ABL, PDGFRβ, AXL, FLT3, RET, c-Src and VEGFR-2. As indicated in Table 3, compounds **10a** and **10b** showed much weaker inhibitory activity against the above kinases. The above results suggested that compounds **10a** and **10b** were potential HGFR and MST1R dual inhibitors. Certainly, only preliminary kinase profile was studied in this work, and further study will be conducted in the following structural modification.

**Table 3.** The kinase inhibitory activity of compounds **10a** and **10b** against ABL, PDGFRβ, AXL, FLT3, RET, c-Src and VEGFR-2.

Compd.	IC_50_ (μM)^a^						
	ABL	RET	AXL	FLT3	PDGFRβ	VEGFR-2	c-Src
**10a**	1.68 ± 0.13	1.27 ± 0.10	0.95 ± 0.086	6.93 ± 0.73	1.13 ± 0.12	1.16 ± 0.11	0.42 ± 0.035
**10b**	3.28 ± 0.26	1.52 ± 0.14	1.50 ± 0.15	>10.0	1.20 ± 0.095	2.36 ± 0.18	0.69 ± 0.057

^a^Values are expressed as the mean of two independent experiments and standard deviations were shown.

### 3.6. Cell apoptosis assay by flow cytometry

**Figure 4.**
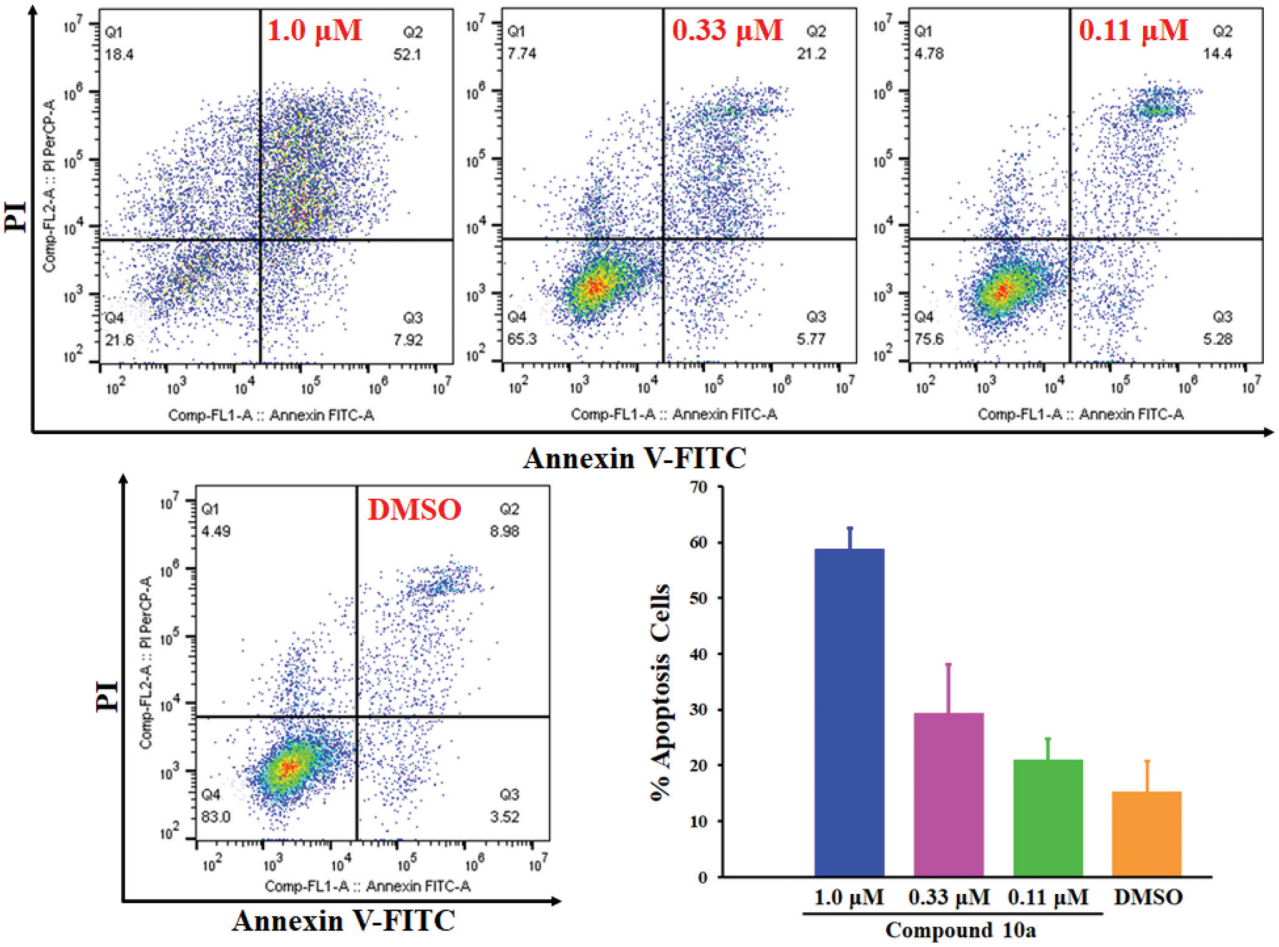
Cell apoptosis analyses of COLO 205 cells treated with compound **10a** for 72 h.

Cell apoptosis assay was conducted to investigate whether the cytotoxic activity of the most potent compound **10a** was caused by the activation of cellular apoptosis in COLO 205 cells. Quantitative analysis of early-apoptotic cells, advanced-apoptotic cells and necrotic cells was determined. COLO 205 cells were stimulated with different concentrations of compound **10a** for 72 h. As depicted in Figure 4, compound **10a** could effectively induce COLO 205 cells apoptosis in a dose-dependent manner. The total apoptosis including early apoptosis and advanced apoptosis accounted for 58.8%, 29.3% and 21.0% (the mean value of three independent determinations) when COLO 205 cells were treated with compound **10a** at the concentration of 1.0 μM, 0.33 μM and 0.11 μM, respectively.

### 3.7. Cell cycle arrest assay by flow cytometry

**Figure 5.**
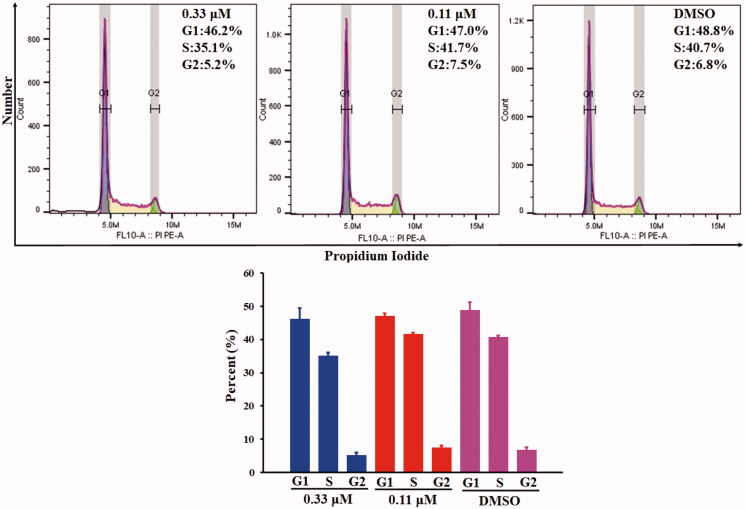
Cell cycle arrest analyses of COLO 205 cells treated with compound **10a** for 24 h.

The antiproliferative activity of compound **10a** was evaluated by flowcytometry analysis of COLO 205 cells. COLO 205 cells were treated with different concentrations (1.0 μM, 0.33 μM and 0.11 μM) of compound **10a** for 24 h. As is shown in Figure 5, compound **10a** could not arrest the cell-cycle progression at the concentration of 0.33 μM and 0.11 μM. At the concentration of 1.0 μM, cell cycle arrest could not be determined due to the massive dead cells (data not shown). The above results indicated that the anticancer mechanism of compound **10a** might be cytotoxicity rather than antiproliferation.

## 4. Experimental

### 4.1. Chemistry

Unless otherwise noted, all chemicals were obtained from commercial vendors and used directly without further purification. Analytical reagent (AR) grade solvents were used for all reactions. Reaction progress was monitored by TLC on pre-coated silica plates (Huanghai HSGF254, 0.20 mm, pH 6.2–6.8) and spots were visualised by UV (254 nm). Flash column chromatography was done using silica gel (Qingdao Ocean Chemical Company, 200–300 mesh). ^1^H NMR and ^13 ^C NMR spectra were recorded on a Bruker AVANCE neo 600. High resolution ESI-MS were recorded on Orbitrap Exploris 240 (Thermo Fisher Science, MA, USA).

#### 4.1.1. 7-(Benzyloxy)-4–(2-fluoro-4-nitrophenoxy)-6-methoxyquinoline (1a)^26^

The mixture of 7-(benzyloxy)-4-chloro-6-methoxyquinoline (21.0 g, 0.07 mol) and 2-fluoro-4-nitrophenol (14.2 g, 0.09 mol) in 120 ml chlorobenzene was refluxed for 14 h. The reaction mixture was cooled to room temperature, and the solvent was concentrated under reduced pressure. The brown residue was dissolved in 300 ml dichloromethane, washed with 10% NaOH aqueous solution (3 × 30 ml) and 50 ml water. The dichloromethane were dried over anhydrous MgSO_4_ and concentrated under reduced pressure to give 21.4 g (yield 72.8%) of the title intermediate as light brown solid. HRMS (m/z), [M + H]^+^ calculated for C_23_H_18_FN_2_O_5_, 421.1200, found, 421.1189.

#### 4.1.2. 7-(Benzyloxy)-N-(2-fluoro-4-nitrophenyl)-6-methoxyquinolin-4-amine (1b)

2-Fluoro-4-nitroaniline (5.7 g, 0.036 mol) and concentrated HCl (cat.) was added to a solution of 7-(benzyloxy)-4-chloro-6-methoxyquinoline (9.0 g, 0.03 mol) in isopropanol, and the reaction mixture was refluxed for 3 h. After completion of the reaction, the mixture was cooled to 0 °C, and the precipitate was filtered off, washed with cold isopropanol, and dried to yield the title intermediate (8.6 g, 68.3%) as a yellow solid. HRMS (m/z), [M + H]^+^ calculated for C_23_H_19_FN_3_O_4_, 420.1360; found, 420.1349.

#### 4.1.3. General procedure for the synthesis of intermediates (2a–b)

Intermediates **1a–b** was dissolved in 33% HBr in acetic acid and the mixtures were stirred for 3 h at room temperature. The precipitate was filtered off, and washed with isopropyl ether to afford target products.

##### 4.1.3.1. *4–(2-Fluoro-4-nitrophenoxy)-6-methoxyquinolin-7-ol (2a)* ^26^

Beige solid, yield: 61.3%. HRMS (m/z), [M + H]^+^ calculated for C_16_H_12_FN_2_O_5_, 331.0730, found, 331.0708.

##### 4.1.3.2. 4-((2-Fluoro-4-nitrophenyl)amino)-6-methoxyquinolin-7-ol (2b)

Yellow solid, yield: 59.5%. ^1^H NMR (600 MHz, DMSO-*d_6_*) δ 9.54 (s, 1H), 9.16 (s, 1H), 8.45 (d, *J* = 5.4 Hz, 1H), 7.74 (m, 1H),7.62–7.65 (m, 1H), 7.48–7.51 (m, 1H), 7.45 (s, 1H), 7.10 (s, 1H), 6.42 (d, *J* = 5.4 Hz, 1H), 3.94 (s, 3H). HRMS (m/z), [M + H]^+^ calculated for C_16_H_13_FN_3_O_4_, 330.0890, found, 330.0861.

#### 4.1.4. General procedure for the synthesis of intermediates (3a–b)

The suspension of **2a–b** (0.05 mol) and Cs_2_CO_3_ (0.12 mol) in DMF (70 ml) was stirred at room temperature for 15 min, and 1-Boc-4-methanesulfonyloxypiperidine (0.075 mol) was added. After stirred at 110 °C for 6 h, the reaction mixture was cooled to room temperature and poured into cold water, filtered, and washed with cold water to give crude products, which were purified by flash chromatography (eluent with 10–20% MeOH in DCM) to afford the title intermediates.

##### 4.1.4.1. Tert-butyl 4-((4–(2-fluoro-4-nitrophenoxy)-6-methoxyquinolin-7-yl)oxy)piperidine-1-carboxylate (3a)

Yellow solid, yield: 57.1%. ^1^H NMR (600 MHz, DMSO-*d_6_*) δ 8.45 (d, *J* = 5.4 Hz, 1H), 7.74 (m, 1H),7.62–7.65 (m, 1H), 7.49 (m, 1H), 7.44 (s, 1H), 7.08 (s, 1H), 6.41 (d, *J* = 5.4 Hz, 1H), 3.93 (s, 3H), 3.82–3.87 (m, 4H), 3.67 (m, 1H), 2.37–2.41 (m, 2H), 2.05–2.07 (m, 2H), 1.44 (s, 9H). HRMS (m/z), [M + H]^+^ calculated for C_26_H_29_FN_3_O_7_, 514.1990, found, 514.1968.

##### 4.1.4.2. Tert-butyl 4-((4-((2-fluoro-4-nitrophenyl)amino)-6-methoxyquinolin-7-yl)oxy)piperidine-1-carboxylate (3b)

Yellow solid, yield: 53.6%. HRMS (m/z), [M + H]^+^ calculated for C_26_H_30_FN_4_O_6_, 513.2149, found, 513.2129.

#### 4.1.5. General procedure for the synthesis of intermediates (4a–b)

The suspension of intermediates **3a–b** (0.02 mol), powered iron (0.06 mol) and concentrated HCl (2 drops) in 90% EtOH (100 ml) was refluxed for 4–6 h with vigorously stirred. After the reaction was completed, the hot mixture was filtered through celites, and the filtrate was evaporated under reduced pressure to afford the title intermediates.

##### 4.1.5.1. *Tert-butyl 4-((4–(4-amino-2-fluorophenoxy)-6-methoxyquinolin-7-yl)oxy)piperidine-1-carboxylate* (*4a)*

Yellow solid, yield: 74.6%. ^1^H NMR (600 MHz, DMSO-*d_6_*) δ 8.44 (d, *J* = 5.4 Hz, 1H), 7.44 (s, 1H), 7.08 (s, 1H), 6.86 (m, 1H), 6.74 (m, 1H), 6.65 (m, 1H), 6.41 (d, *J* = 5.4 Hz, 1H), 5.74 (br, 2H), 3.93 (s, 3H), 3.83–3.87 (m, 4H), 3.67 (m, 1H), 2.37–2.41 (m, 2H), 2.05–2.08 (m, 2H), 1.44 (s, 9H). HRMS (m/z), [M + H]^+^ calculated for C_26_H_31_FN_3_O_5_, 484.2248, found, 484.2227.

##### 4.1.5.2. Tert-butyl 4-((4-((4-amino-2-fluorophenyl)amino)-6-methoxyquinolin-7-yl)oxy)piperidine-1-carboxylate (4b)

Yellow solid, yield: 77.8%. HRMS (m/z), [M + H]^+^ calculated for C_26_H_32_FN_4_O_4_, 483.2408, found, 483.2385.

#### 4.1.6. General procedure for the synthesis of intermediates (6a–b)

Phenyl chloroformate (20.0 mmol) was added to a solution of amines **4a–b** (10.0 mmol) and dry pyridine (30.0 mmol) in dry CH_2_Cl_2_ (40 ml) at 0 °C. After the addition was completed, the mixture was allowed to warm to room temperature for another 2 h, and then saturated NaHCO_3_ aqueous solution was added to the solution. The CH_2_Cl_2_ phase was separated, washed with water, dried over anhydrous MgSO_4_, and concentrated under reduced pressure to afford intermediates **5a–b**, which were immediately used in the following step without further purification.

To a mixture of esters **5a–b** in 20 ml xylene was added hydrazine monohydrate (50%, 20 ml). The reaction mixture was stirred vigorously at 70 °C for 2 h. The solvent and excessive hydrazine monohydrate were evaporated under reduced pressure, and the residue was purified by flash chromatography (eluent with 1–10% MeOH in DCM, 1% Et_3_N) to afford semicarbazides **6a–b**.

##### 4.1.6.1. *Tert-butyl 4-((4–(2-fluoro-4-(hydrazinecarboxamido)phenoxy)-6-methoxyquinolin-7-yl)oxy)piperidine-1-carboxylate* (*6a)*

Yellow solid, yield: 35.4%. ^1^H NMR (600 MHz, DMSO-*d_6_*) δ 9.19 (s, 1H), 8.83 (s, 1H), 8.44 (d, *J* = 5.4 Hz, 1H), 7.64 (m, 1H), 7.44 (s, 1H), 7.15 (m, 1H), 7.08 (s, 1H), 6.86 (m, 1H), 6.41 (d, *J* = 5.4 Hz, 1H), 5.74 (br, 2H), 4.62 (s, 2H), 3.95 (s, 3H), 3.83–3.87 (m, 4H), 3.65 (m, 1H), 2.37–2.41 (m, 2H), 2.05–2.08 (m, 2H), 1.42 (s, 9H). HRMS (m/z), [M + H]^+^ calculated for C_27_H_33_FN_5_O_6_, 542.2415, found, 542.2387.

##### 4.1.6.2. Tert-butyl 4-((4-((2-fluoro-4-(hydrazinecarboxamido)phenyl)amino)-6-methoxyquinolin-7-yl)oxy)piperidine-1-carboxylate (6b)

Yellow solid, yield: 39.1%. HRMS (m/z), [M + H]^+^ calculated for C_27_H_34_FN_6_O_5_, 541.2575, found, 541.2554.

#### 4.1.7. General procedure for the synthesis of intermediates (7a–b)

The mixture of **6a–h** (10.0 mmol), 2,6-difluorobenzaldehyde (12.0 mmol) and acetic acid (cat.) in dry *i*-PrOH (50 ml) was refluxed for 2–3 h. The mixture was cooled to 0 °C, and the resultant precipitate was filtered, washed with cold *i*-PrOH and dried *in vacuo* to give semicarbazones **7a–b**.

##### 4.1.7.1. Tert-butyl 4-((4–(4-(2–(2,6-difluorobenzylidene)hydrazine-1-carboxamido)-2-fluorophenoxy)-6-methoxyquinolin-7-yl)oxy)piperidine-1-carboxylate (7a)

White solid, yield: 80.1%. HRMS (m/z), [M + H]^+^ calculated for C_34_H_35_F_3_N_5_O_6_, 666.2539, found, 666.2518.

##### 4.1.7.2. Tert-butyl 4-((4-((4–(2–(2,6-difluorobenzylidene)hydrazine-1-carboxamido)-2-fluorophenyl)amino)-6-methoxyquinolin-7-yl)oxy)piperidine-1-carboxylate (7b)

Light yellow solid, yield: 75.4%. HRMS (m/z), [M + H]^+^ calculated for C_34_H_36_F_3_N_6_O_5_, 665.2699, found, 665.2676.

#### 4.1.8. General procedure for the synthesis of intermediates (8a–b)

The solution of **7a–b** (0.015 mol) and CF_3_COOH (0.15 mol) in CH_2_Cl_2_ (50 ml) was stirred for 2 h at room temperature Evaporation of the solvent and excessive CF_3_COOH provided yellow oil. The residue was diluted with 100 ml CH_2_Cl_2_, and 20% NaOH aqueous solution was added until the pH reached to 9. The organic phase was washed with water, dried over anhydrous MgSO_4_, concentrated *in vacuo*, and the residue was used for the next step without further purification.

##### 4.1.8.1. 2-(2,6-Difluorobenzylidene)-N-(3-fluoro-4-((6-methoxy-7-(piperidin-4-yloxy)quinolin-4-yl)oxy)phenyl)hydrazine-1-carboxamide (8a)

Light yellow solid, yield: 83.7%. ^1^H NMR (600 MHz, DMSO-*d_6_*) δ 8.92 (s, 1H), 8.83 (s, 1H), 8.44 (d, *J* = 5.4 Hz, 1H), 8.31 (s, 1H), 7.62 (m, 1H), 7.44 (s, 1H), 7.31 (m, 1H), 7.12–7.16 (m, 3H), 7.08 (s, 1H), 6.86 (m, 1H), 6.41 (d, *J* = 5.4 Hz, 1H), 3.95 (s, 3H), 3.83–3.87 (m, 4H), 3.65 (m, 1H), 2.37–2.41 (m, 2H), 2.05–2.08 (m, 2H), 1.96 (br, 1H). HRMS (m/z), [M + H]^+^ calculated for C_29_H_27_F_3_N_5_O_4_, 566.2015; found, 566.1987.

##### 4.1.8.2. 2–(2,6-Difluorobenzylidene)-N-(3-fluoro-4-((6-methoxy-7-(piperidin-4-yloxy)quinolin-4-yl)amino)phenyl)hydrazine-1-carboxamide (8b)

Light yellow solid, yield: 81.4%. HRMS (m/z), [M + H]^+^ calculated for C_29_H_28_F_3_N_6_O_3_, 565.2175, found, 565.2149.

#### 4.1.9. General procedure for the synthesis of intermediates (9a–h)

Method A for the preparation of **9b**, **9d**, **9e** and **9g**. To a cold solution of **8a** (0.01 mol) and Et_3_N (0.015 mmol) in dry 30 ml CH_2_Cl_2_ was added acyl chloride (0.013 mol) dropwise. Then, the reaction mixture was stirred at room temperature for 4–5 h, saturated NaHCO_3_ aqueous solution was added, and the CH_2_Cl_2_ phase was separated, dried over anhydrous MgSO_4_, and concentrated in vacuum to afford the title intermediates.

Method B for the preparation of **9a**, **9c**, **9f** and **9h**. To a suspension of **8a–b** (0.01 mol) and Cs_2_CO_3_ (0.015 mol) in 30 ml DMF was added 2-chloroamides (0.015 mol). The resulting mixture was stirred for 6–8 h at 90 °C, and then poured into cold water. The precipitate was filtered off, washed with water, and dried to afford the title intermediate.

##### 4.1.9.1. N-(4-((7-((1–(2-amino-2-oxoethyl)piperidin-4-yl)oxy)-6-methoxyquinolin-4-yl)oxy)-3-fluorophenyl)-2–(2,6-difluorobenzylidene)hydrazine-1-carboxamide (9a)

Yellow solid, yield: 68.8%. ^1^H NMR (600 MHz, DMSO-*d_6_*) δ 9.14 (s, 1H), 8.91 (s, 1H), 8.43 (d, *J* = 5.4 Hz, 1H), 8.26 (s, 1H), 7.62–7.65 (m, 1H), 7.53 (s, 1H), 7.48–7.51 (m, 1H), 7.45 (s, 1H), 7.34–7.37 (m, 1H), 7.26 (m, 1H), 7.21 (m, 1H), 7.16–7.19 (m, 1H), 7.10 (m, 1H), 6.45 (d, *J* = 5.4 Hz, 1H), 4.62–4.65 (m, 1H), 3.93 (s, 3H), 2.89 (s, 2H), 2.75–2.76 (m, 2H), 2.37–2.41 (m, 2H), 2.04–2.07 (m, 2H), 1.76–1.81 (m, 2H). HRMS (m/z), [M + H]^+^ calculated for C_31_H_30_F_3_N_6_O_5_, 623.2230, found, 623.2205.

##### 4.1.9.2. 2–(2,6-Difluorobenzylidene)-N-(3-fluoro-4-((6-methoxy-7-((1–(2-methoxyacetyl)piperidin-4-yl)oxy)quinolin-4-yl)oxy)phenyl)hydrazine-1-carboxamide (9b)

Yellow solid, yield: 79.7%. ^1^H NMR (600 MHz, DMSO-*d_6_*) δ 9.22 (s, 1H), 8.87 (s, 1H), 8.46 (d, *J* = 5.4 Hz, 1H), 8.25 (s, 1H), 7.62–7.65 (m, 1H), 7.55 (s, 1H), 7.52 (s, 1H), 7.48–7.53 (m, 1H), 7.34–7.37 (m, 1H), 7.27 (m, 1H), 7.16–7.19 (m, 1H), 6.44 (d, *J* = 5.4 Hz, 1H), 4.88–4.91 (m, 1H), 4.52 (br, 1H), 4.13 (s, 2H), 3.94 (s, 3H), 3.62–3.64 (m, 1H), 3.32 (s, 3H), 3.05–3.10 (m, 2H), 2.00–2.05 (m, 2H), 1.64–1.72 (m, 2H). HRMS (m/z), [M + H]^+^ calculated for C_32_H_31_F_3_N_5_O_6_, 638.2226, found, 638.2203.

##### 4.1.9.3. 2–(2,6-Difluorobenzylidene)-N-(3-fluoro-4-((6-methoxy-7-((1–(2-(methylamino)-2-oxoethyl)piperidin-4-yl)oxy)quinolin-4-yl)oxy)phenyl)hydrazine-1-carboxamide (9c)

Yellow solid, yield: 59.7%. HRMS (m/z), [M + H]^+^ calculated for C_32_H_32_F_3_N_6_O_5_, 637.2386, found, 637.2357.

##### 4.1.9.4. 2–(2,6-Difluorobenzylidene)-N-(4-((7-((1-(dimethylglycyl)piperidin-4-yl)oxy)-6-methoxyquinolin-4-yl)oxy)-3-fluorophenyl)hydrazine-1-carboxamide (9d)

Yellow solid, yield: 69.4%. HRMS (m/z), [M + H]^+^ calculated for C_33_H_34_F_3_N_6_O_5_, 651.2543, found, 651.2522.

##### 4.1.9.5. 2–(2,6-Difluorobenzylidene)-N-(3-fluoro-4-((6-methoxy-7-((1-(morpholine-4-carbonyl)piperidin-4-yl)oxy)quinolin-4-yl)oxy)phenyl)hydrazine-1-carboxamide (9e)

Yellow solid, yield: 66.3%. ^1^H NMR (600 MHz, DMSO-*d_6_*) δ 9.10 (s, 1H), 8.91 (s, 1H), 8.44 (d, *J* = 5.4 Hz, 1H), 8.27 (s, 1H), 7.62–7.65 (m, 1H), 7.55 (s, 1H), 7.52 (s, 1H), 7.48–7.50 (m, 2H), 7.34–7.37 (m, 1H), 7.26 (m, 1H), 7.16–7.19 (m, 2H), 6.42 (d, *J* = 5.4 Hz, 1H), 4.81–4.85 (m, 1H), 3.95 (s, 3H), 3.57 (m, 4H), 3.49–3.52 (m, 2H), 3.13–3.16 (m, 4H), 3.09–3.12 (m, 2H), 2.01–2.05 (m, 2H), 1.65–1.70 (m, 2H). HRMS (m/z), [M + H]^+^ calculated for C_34_H_34_F_3_N_6_O_6_, 679.2492, found, 679.2471.

##### 4.1.9.6. 2–(2,6-Difluorobenzylidene)-N-(3-fluoro-4-((6-methoxy-7-((1–(2-morpholino-2-oxoethyl)piperidin-4-yl)oxy)quinolin-4-yl)oxy)phenyl)hydrazine-1-carboxamide (9f)

Yellow solid, yield: 55.9%. HRMS (m/z), [M + H]^+^ calculated for C_35_H_36_F_3_N_6_O_6_, 693.2648, found, 693.2627.

##### 4.1.9.7. 2–(4-((4–(4-(2–(2,6-Difluorobenzylidene)hydrazine-1-carboxamido)-2-fluorophenoxy)-6-methoxyquinolin-7-yl)oxy)piperidin-1-yl)-2-oxoethyl acetate (9g)

Yellow solid, yield: 65.5%. ^1^H NMR (600 MHz, DMSO-*d_6_*) δ 9.22 (s, 1H), 8.76 (s, 1H), 8.45 (d, *J* = 5.4 Hz, 1H), 8.26 (s, 1H), 7.63–7.65 (m, 1H), 7.55 (s, 1H), 7.53 (s, 1H), 7.48–7.53 (m, 1H), 7.34–7.37 (m, 1H), 7.26 (m, 1H), 7.16–7.19 (m, 2H), 6.45 (d, *J* = 5.4 Hz, 1H), 4.88–4.90 (m, 1H), 4.52 (s, 1H), 4.13 (s, 2H), 3.95 (m, 4H), 3.62 (m, 1H), 3.08 (m, 1H), 2.26 (s, 3H), 2.05 (m, 2H), 1.62–1.71 (m, 2H). HRMS (m/z), [M + H]^+^ calculated for C_33_H_31_F_3_N_5_O_7_, 666.2176, found, 666.2148.

##### 4.1.9.8. N-(4-((7-((1–(2-amino-2-oxoethyl)piperidin-4-yl)oxy)-6-methoxyquinolin-4-yl)amino)-3-fluorophenyl)-2–(2,6-difluorobenzylidene)hydrazine-1-carboxamide (9h)

Yellow solid, yield: 52.4%. HRMS (m/z), [M + H]^+^ calculated for C_31_H_31_F_3_N_7_O_4_, 622.2390, found, 622.2369.

#### 4.1.10. General procedure for the synthesis of target compounds 10a–g and 10i

To a suspension of intermediates **9a–h** (0.3 mmol) in dry CH_2_Cl_2_ (5 ml), mercaptoacetic acid (0.3 ml) and SiCl_4_ (15 drops) were added subsequently at 0 °C. The reaction mixture was allowed to cooled to room temperature, and refluxed for 6–8 h. The mixture was cooled to room temperature, and quenched by 2 ml cold water. After stirred for 5 min, 10% NaOH aqueous solution was added until pH reached to 10. The CH_2_Cl_2_ phase was separated and washed with water (2 × 5 ml), concentrated under reduced pressure to yield crude products which were purified by flash chromatography (eluent with 5–10% MeOH in DCM, 1% Et_3_N) to give target compounds.

##### 4.1.10.1. 2–(4-((4–(4-(3–(2-(2,6-Difluorophenyl)-4-oxothiazolidin-3-yl)ureido)-2-fluorophenoxy)-6-methoxyquinolin-7-yl)oxy)piperidin-1-yl)acetamide (10a)

White solid, yield: 32.7%. HPLC purity: 99.75%. ^1^H NMR (600 MHz, DMSO-*d_6_*) δ 9.20 (s, 1H), 8.93 (s, 1H), 8.45 (d, *J* = 5.4 Hz, 1H), 7.62–7.65 (m, 1H), 7.53 (s, 1H), 7.48–7.51 (m, 1H), 7.45 (s, 1H), 7.34–7.37 (m, 1H), 7.26 (m, 1H), 7.21 (m, 1H), 7.16–7.19 (m, 1H), 7.10 (m, 1H), 6.42 (d, *J* = 5.4 Hz, 1H), 6.16 (s, 1H), 4.62–4.65 (m, 1H), 3.94 (s, 3H), 3.81–3.87 (m, 2H), 2.89 (s, 2H), 2.75–2.77 (m, 2H), 2.37–2.41 (m, 2H), 2.05–2.07 (m, 2H), 1.76–1.82 (m, 2H). ^13 ^C NMR (101 MHz, DMSO-*d_6_*) δ 171.8, 168.4, 161.6, 159.9, 159.3, 154.3, 153.6, 152.7, 150.1, 150.1, 148.7, 146.2, 138.2, 138.1, 134.7, 134.6, 131.5, 124.0, 115.1, 114.5, 112.3, 110.2, 101.8, 99.3, 73.0, 61.1, 55.7, 51.8, 50.8 (2 C), 30.2 (2 C), 29.2. HRMS (m/z), [M + H]^+^ calculated for C_33_H_32_F_3_N_6_O_6_S, 697.2056, found, 697.2023.

##### 4.1.10.2. 1–(2-(2,6-Difluorophenyl)-4-oxothiazolidin-3-yl)-3–(3-fluoro-4-((6-methoxy-7-((1–(2-methoxyacetyl)piperidin-4-yl)oxy)quinolin-4-yl)oxy)phenyl)urea (10b)

White solid, yield: 29.4%. ^1^H NMR (600 MHz, DMSO-*d_6_*) δ 9.29 (s, 1H), 8.93 (s, 1H), 8.46 (d, *J* = 5.4 Hz, 1H), 7.62–7.65 (m, 1H), 7.55 (s, 1H), 7.54 (s, 1H), 7.48–7.53 (m, 1H), 7.34–7.37 (m, 1H), 7.27 (m, 1H), 7.16–7.19 (m, 1H), 6.44 (d, *J* = 5.4 Hz, 1H), 6.16 (s, 1H), 4.88–4.91 (m, 1H), 4.52 (br, 1H), 4.13 (s, 2H), 3.95 (s, 3H), 3.81–3.87 (m, 2H), 3.62–3.64 (m, 1H), 3.33 (s, 3H), 3.05–3.10 (m, 2H), 2.00–2.05 (m, 2H), 1.62–1.72 (m, 2H). ^13 ^C NMR (101 MHz, DMSO-*d_6_*) δ 169.3, 167.9, 161.0, 159.4, 158.9, 153.8, 153.2, 152.1, 149.6, 149.4, 148.3, 145.5, 137.7, 137.7, 134.1, 134.0, 131.0, 123.5, 114.5, 114.1, 111.8, 109.9, 101.4, 98.9, 72.2, 59.4, 55.3, 51.2, 44.9 (2 C), 40.2, 30.0, 29.4, 28.7. HRMS (m/z), [M + H]^+^ calculated for C_34_H_33_F_3_N_5_O_7_S, 712.2053; found, 712.2039.

##### 4.1.10.3. 2–(4-((4–(4-(3–(2-(2,6-Difluorophenyl)-4-oxothiazolidin-3-yl)ureido)-2-fluorophenoxy)-6-methoxyquinolin-7-yl)oxy)piperidin-1-yl)-N-methylacetamide (10c)

White solid, yield: 30.8%. ^1^H NMR (600 MHz, DMSO-*d_6_*) δ 9.34 (s, 1H), 8.46 (d, *J* = 5.4 Hz, 1H), 8.15–8.17 (m, 1H), 7.85–7.83 (m, 1H), 7.72 (m, 1H), 7.55 (s, 1H), 7.50–7.53 (m, 1H), 7.46 (s, 1H), 7.42–7.45 (m, 2H), 7.22–7.25 (m, 2H), 6.45 (d, *J* = 5.4 Hz, 1H), 6.16 (s, 1H), 4.63–4.65 (m, 1H), 3.95 (s, 3H), 3.81–3.84 (m, 2H), 2.93 (s, 2H), 2.75–2.77 (m, 2H), 2.64 (s, 3H), 2.37–2.41 (m, 2H), 2.05–2.07 (m, 2H), 1.79–1.84 (m, 2H). HRMS (m/z), [M + H]^+^ calculated for C_34_H_34_F_3_N_6_O_6_S, 711.2213; found, 711.2184.

##### 4.1.10.4. 1–(2-(2,6-Difluorophenyl)-4-oxothiazolidin-3-yl)-3–(4-((7-((1-(dimethylglycyl)piperidin-4-yl)oxy)-6-methoxyquinolin-4-yl)oxy)-3-fluorophenyl)urea (10d)

White solid, yield: 31.4%. ^1^H NMR (600 MHz, DMSO-*d_6_*) δ 9.17 (s, 1H), 8.91 (s, 1H), 8.45 (d, *J* = 5.4 Hz, 1H), 7.61–7.65 (m, 1H), 7.54 (s, 1H), 7.53 (s, 1H), 7.48–7.52 (m, 1H), 7.34–7.37 (m, 1H), 7.26–7.28 (m, 1H), 7.16–7.19 (m, 1H), 6.42 (d, *J* = 5.4 Hz, 1H), 6.16 (1H), 4.87–4.90 (m, 1H), 3.94 (s, 3H), 3.81–3.89 (m, 3H), 3.41–3.44 (m, 1H), 3.24–3.26 (m, 2H), 3.08–3.16 (m, 2H), 2.21 (s, 6H), 1.98–2.08 (m, 2H), 1.70 (m, 1H), 1.58(m, 1H). ^13 ^C NMR (101 MHz, DMSO-*d_6_*) δ 168.5, 167.6, 161.6, 160.0, 159.4, 153.7, 152.7, 152.2, 150.1, 148.8, 146.2, 138.2, 138.2, 134.7, 134.7, 131.5, 124.0, 115.1, 114.8, 114.7, 112.4, 110.5, 101.9, 99.5, 72.9, 61.9, 55.8, 51.7, 45.1 (2 C), 42.3, 38.5, 31.0, 30.2, 29.2. HRMS (m/z), [M + H]^+^ calculated for C_35_H_36_F_3_N_6_O_6_S, 725.2369; found, 725.2345.

##### 4.1.10.5. 1–(2-(2,6-Difluorophenyl)-4-oxothiazolidin-3-yl)-3–(3-fluoro-4-((6-methoxy-7-((1-(piperidine-1-carbonyl)piperidin-4-yl)oxy)quinolin-4-yl)oxy)phenyl)urea (10e)

White solid, yield: 33.6%. ^1^H NMR (600 MHz, DMSO-*d_6_*) δ 9.16 (s, 1H), 8.90 (s, 1H), 8.45 (d, *J* = 5.4 Hz, 1H), 7.62–7.65 (m, 1H), 7.54 (s, 1H), 7.52 (s, 1H), 7.48–7.50 (m, 2H), 7.34–7.37 (m, 1H), 7.26 (m, 1H), 7.16–7.19 (m, 2H), 6.42 (d, *J* = 5.4 Hz, 1H), 6.16 (s, 1H), 4.81–4.85 (m, 1H), 3.94 (s, 3H), 3.81–3.87 (m, 2H), 3.57 (m, 4H), 3.49–3.52 (m, 2H), 3.13–3.16 (m, 4H), 3.10–3.12 (m, 2H), 2.01–2.05 (m, 2H), 1.65–1.71 (m, 2H). ^13 ^C NMR (101 MHz, DMSO-*d_6_*) δ 167.9, 162.5, 161.0, 159.4, 158.8, 153.8, 153.1, 152.1, 149.6, 148.3, 145.6, 137.7, 137.6, 134.2, 134.1, 131.0, 123.5, 114.6, 114.2, 114.1, 111.7, 110.0, 101.4, 98.9, 72.6, 65.3, 55.3 (2 C), 51.1, 46.5 (2 C), 43.0 (2 C), 29.6 (2 C), 28.7. HRMS (m/z), [M + H]^+^ calculated for C_37_H_38_F_3_N_6_O_6_S, 753.2318; found, 753.2291.

##### 4.1.10.6. 1–(2-(2,6-Difluorophenyl)-4-oxothiazolidin-3-yl)-3–(3-fluoro-4-((6-methoxy-7-((1–(2-morpholino-2-oxoethyl)piperidin-4-yl)oxy)quinolin-4-yl)oxy)phenyl)urea (10f)

White solid, yield: 30.8%. ^1^H NMR (600 MHz, DMSO-*d_6_*) δ 9.21(s, 1H), 8.93 (s, 1H), 8.45 (d, *J* = 5.4 Hz, 1H), 7.62–7.65 (m, 1H), 7.53 (s, 1H), 7.48–7.51 (m, 1H), 7.45 (s, 1H), 7.34–7.37 (m, 1H), 7.26 (m, 1H), 7.16–7.19 (m, 2H), 6.41 (d, *J* = 5.4 Hz, 1H), 6.16 (s, 1H), 4.60–4.63 (m, 1H), 3.94 (s, 3H), 3.81–3.87 (m, 2H), 3.59 (m, 4H), 3.54 (m, 2H), 3.44 (m, 2H), 3.19 (s, 2H), 2.75 (m, 2H), 2.35–2.38 (m, 2H), 2.04–2.06 (m, 2H), 1.69–1.74 (m, 2H). ^13 ^C NMR (101 MHz, DMSO-*d_6_*) δ 167.9, 167.2, 161.0, 159.4, 158.8, 153.8, 153.1, 152.1, 149.6, 148.2, 145.7, 137.7, 137.6, 134.2, 134.1, 131.0, 123.5, 114.5, 114.2, 114.0, 111.8, 109.7, 101.3, 98.8, 72.5, 65.9, 65.7, 59.8, 55.2 (2 C), 51.2, 49.6 (2 C), 45.3 (2 C), 41.1, 29.9 (2 C), 28.7. HRMS (m/z), [M + H]^+^ calculated for C_37_H_38_F_3_N_6_O_7_S, 767.2475; found, 767.2445.

##### 4.1.10.7. 2–(4-((4–(4-(3–(2-(2,6-Difluorophenyl)-4-oxothiazolidin-3-yl)ureido)-2-fluorophenoxy)-6-methoxyquinolin-7-yl)oxy)piperidin-1-yl)-2-oxoethyl acetate (10g)

White solid, yield: 31.6%. ^1^H NMR (600 MHz, DMSO-*d_6_*) δ 9.28 (s, 1H), 8.92 (s, 1H), 8.46 (d, *J* = 5.4 Hz, 1H), 7.63–7.65 (m, 1H), 7.55 (s, 1H), 7.54 (s, 1H), 7.48–7.53 (m, 1H), 7.34–7.37 (m, 1H), 7.26 (m, 1H), 7.16–7.19 (m, 2H), 6.43 (d, *J* = 5.4 Hz, 1H), 6.16 (s, 1H), 4.88–4.90 (m, 1H), 4.52 (s, 1H), 4.13 (s, 2H), 3.95 (m, 4H), 3.81–3.87 (m, 2H), 3.62 (m, 1H), 3.08 (m, 1H), 2.27 (s, 3H), 2.05 (m, 2H), 1.62–1.72 (m, 2H). ^13 ^C NMR (101 MHz, DMSO-*d_6_*) δ 170.4, 169.0, 162.1, 160.5, 159.9, 154.8, 154.2, 153.2, 150.7, 150.5, 149.3, 146.6, 138.8, 138.7, 135.2, 135.1, 132.0, 124.6, 115.6, 115.2, 112.9, 111.0, 102.5, 100.0, 72.3, 60.5, 56.3, 52.2, 46.0 (2 C), 40.4, 31.0, 30.5, 29.7. HRMS (m/z), [M + H]^+^ calculated for C_35_H_33_F_3_N_5_O_8_S, 740.2002; found, 740.1979.

##### 4.1.10.8. 2–(4-((4-((4–(3-(2–(2,6-Difluorophenyl)-4-oxothiazolidin-3-yl)ureido)-2-fluorophenyl)amino)-6-methoxyquinolin-7-yl)oxy)piperidin-1-yl)acetamide (10i)

White solid, yield: 31.9%. ^1^H NMR (600 MHz, DMSO-*d_6_*) δ 9.21 (s, 1H), 9.12 (s, 1H), 8.93 (s, 1H), 8.46 (d, *J* = 5.4 Hz, 1H), 7.62–7.65 (m, 1H), 7.54 (s, 1H), 7.48–7.51 (m, 1H), 7.46 (s, 1H), 7.34–7.37 (m, 1H), 7.26 (m, 1H), 7.21 (m, 1H), 7.16–7.19 (m, 1H), 7.10 (m, 1H), 6.43 (d, *J* = 5.4 Hz, 1H), 6.16 (s, 1H), 4.62–4.65 (m, 1H), 3.93 (s, 3H), 3.81–3.87 (m, 2H), 2.88 (s, 2H), 2.75–2.77 (m, 2H), 2.37–2.40 (m, 2H), 2.04–2.07 (m, 2H), 1.76–1.82 (m, 2H). HRMS (m/z), [M + H]^+^ calculated for C_33_H_33_F_3_N_7_O_5_S, 696.2216; found, 696.2187.

#### 4.1.11. 1–(2-(2,6-Difluorophenyl)-4-oxothiazolidin-3-yl)-3–(3-fluoro-4-((7-((1–(2-hydroxyacetyl)piperidin-4-yl)oxy)-6-methoxyquinolin-4-yl)oxy)phenyl)urea (10h)

To a suspension of compound **10g** (30 mg, 0.04 mmol) in 1 ml MeOH, NaOH solution (4.8 mg in 0.5 ml H_2_O) was added. After stirred for 1 h at 50 °C, the solution was concentrated under reduced pressure, and then 1 ml water was added to the residue. Concentrated HCl was added cautiously until pH reached to 4–5, and the resultant precipitate was filtered, washed with water and dried *in vacuo* to give the title compound as a white solid, yield: 75.9%. ^1^H NMR (600 MHz, DMSO-*d_6_*) δ 9.24 (s, 1H), 8.93 (s, 1H), 8.45 (d, *J* = 5.4 Hz, 1H), 7.63–7.65 (m, 1H), 7.56 (s, 1H), 7.54 (s, 1H), 7.48–7.53 (m, 1H), 7.34–7.37 (m, 1H), 7.25 (m, 1H), 7.16–7.19 (m, 2H), 6.43 (d, *J* = 5.4 Hz, 1H), 6.16 (s, 1H), 4.88–4.90 (m, 1H), 4.75 (br, 1H), 4.52 (s, 1H), 4.12 (s, 2H), 3.95 (m, 4H), 3.81–3.87 (m, 2H), 3.62 (m, 1H), 3.07 (m, 1H), 2.05 (m, 2H), 1.62–1.72 (m, 2H). HRMS (m/z), [M + H]^+^ calculated for C_33_H_31_F_3_N_5_O_7_S, 698.1896, found, 698.1871.

#### 4.1.12. 7-(Benzyloxy)-4–(2-fluoro-4-nitrophenoxy)-6-methoxyquinazoline (11)

The mixture of 7-(benzyloxy)-4-chloro-6-methoxyquinazoline (9.0 g, 0.03 mol) and 2-fluoro-4-nitrophenol (6.3 g, 0.04 mol) in 60 ml chlorobenzene was refluxed for 13 h. The reaction mixture was cooled to room temperature, and the solvent was evaporated under reduced pressure. The residue was dissolved in 300 ml CH_2_Cl_2_, washed with 10% NaOH aqueous solution (3 × 30 ml) and 50 ml water. The CH_2_Cl_2_ phase was dried over anhydrous MgSO_4_ and concentrated under reduced pressure to give 8.2 g (yield 64.9%) of the title intermediate as dark yellow solid. HRMS (m/z), [M + H]^+^ calculated for C_22_H_17_FN_3_O_5_, 422.1152; found, 422.1128.

#### 4.1.13. 4–(2-Fluoro-4-nitrophenoxy)-6-methoxyquinazolin-7-ol (12)

Intermediate **11** (8.2 g, 0.019 mol) was dissolved in 33% HBr in acetic acid (45 ml) and the mixtures were stirred for 3 h at room temperature. The precipitate was filtered off, and washed with isopropyl ether to afford target products as beige solid (4.1 g, 64.1%). ^1^H NMR (600 MHz, DMSO-*d_6_*) δ 9.43 (s, 1H), 8.59 (s 1H), 7.74 (m, 1H),7.62–7.65 (m, 1H), 7.45 (s, 1H), 7.32 (s, 1H), 7.10 (m, 1H), 3.92 (s, 3H). HRMS (m/z), [M + H]^+^ calculated for C_15_H_11_FN_3_O_5_, 332.0683; found, 332.0662.

#### 4.1.14. Tert-butyl 4-((4–(2-fluoro-4-nitrophenoxy)-6-methoxyquinazolin-7-yl)oxy)piperidine-1-carboxylate (13)

The suspension of **12** (4.1 g, 0.012 mol) and Cs_2_CO_3_ (8.2 g, 0.025 mol) in DMF (25 ml) was stirred at room temperature for 15 min, and 1-Boc-4-methanesulfonyloxypiperidine (5.0 g, 0.018 mol) was added. After stirred at 110 °C for 6 h, the reaction mixture was cooled to room temperature and poured into ice water, filtered, and washed with cold water to give crude products, which were purified by flash chromatography (eluent with 10–20% MeOH in DCM) to afford the title intermediate **13** as a yellow solid (3.0 g, 49.6%). HRMS (m/z), [M + H]^+^ calculated for C_25_H_28_FN_4_O_7_, 515.1942; found, 515.1921.

#### 4.1.15. Tert-butyl 4-((4–(4-amino-2-fluorophenoxy)-6-methoxyquinazolin-7-yl)oxy)piperidine-1-carboxylate (14)

The suspension of intermediate **13** (0.02 mol), powered iron (0.06 mol) and concentrated HCl (2 drops) in 90% EtOH (100 ml) was refluxed for 4–6 h with vigorously stirred. After the reaction was completed, the hot mixture was filtered through celites, and the filtrate was evaporated under reduced pressure to afford the title intermediate as dark yellow solid (6.9 g, 71.2%). ^1^H NMR (600 MHz, DMSO-*d_6_*) δ 8.57 (s, 1H), 7.44 (s, 1H), 7.08 (s, 1H), 6.86 (m, 1H), 6.74 (m, 1H), 6.65 (m, 1H), 5.64 (s, 2H), 3.95 (s, 3H), 3.83–3.87 (m, 4H), 3.65 (m, 1H), 2.37–2.41 (m, 2H), 2.05–2.08 (m, 2H), 1.43 (s, 9H). HRMS (m/z), [M + H]^+^ calculated for C_20_H_20_FN_4_O_5_, 485.2200; found, 485.2178.

#### 4.1.16. Tert-butyl 4-((4–(2-fluoro-4-(hydrazinecarboxamido)phenoxy)-6-methoxyquinazolin-7-yl)oxy)piperidine-1-carboxylate (15)

Phenyl chloroformate (28.2 mmol) was added to a solution of amine **14** (6.9 g, 14.2 mmol) and dry pyridine (42.6 mmol) in dry CH_2_Cl_2_ (350 ml) at 0 °C. After the addition was completed, the mixture was allowed to warm to room temperature for another 2 h, and then saturated NaHCO_3_ aqueous solution was added to the solution. The CH_2_Cl_2_ phase was separated, washed with water, dried over anhydrous MgSO_4_, and concentrated under reduced pressure to afford brown oil, which were immediately used in the following step without further purification.

The above oil was dissolved in 20 ml xylene was added hydrazine monohydrate (50%, 20 ml). The reaction mixture was stirred vigorously at 70 °C for 2 h. The solvent and excessive hydrazine monohydrate were evaporated under reduced pressure, and the residue was purified by flash chromatography (eluent with 1–10% MeOH in DCM, 1% Et_3_N) to afford semicarbazide **15** as a light yellow solid (2.7 g, 35.5%). HRMS (m/z), [M + H]^+^ calculated for C_26_H_32_FN_6_O_6_, 543.2367; found, 543.2346.

#### 4.1.17. Tert-butyl 4-((4–(4-(2–(2,6-difluorobenzylidene)hydrazine-1-carboxamido)-2-fluorophenoxy)-6-methoxyquinazolin-7-yl)oxy)piperidine-1-carboxylate (16)

The mixture of **15** (2.0 g, 3.7 mmol), 2,6-difluorobenzaldehyde (4.4 mmol) and acetic acid (cat.) in dry *i*-PrOH (12 ml) was refluxed for 3 h. The mixture was cooled to 0 °C, and the resultant precipitate was filtered, washed with cold *i*-PrOH and dried in vacuum to give semicarbazone **16** as a beige solid (1.8 g, 75.6%). HRMS (m/z), [M + H]^+^ calculated for C_33_H_34_F_3_N_6_O_6_, 667.2492; found, 667.2471.

#### 4.1.18. 2–(2,6-Difluorobenzylidene)-N-(3-fluoro-4-((6-methoxy-7-(piperidin-4-yloxy)quinazolin-4-yl)oxy)phenyl)hydrazine-1-carboxamide (17)

The solution of **16** (1.5 g, 2.2 mmol) and CF_3_COOH (22.0 mmol) in CH_2_Cl_2_ (10 ml) was stirred for 2 h at room temperature Evaporation of the solvent and excessive CF_3_COOH provided yellow oil. The residue was diluted with 20 ml CH_2_Cl_2_, and 20% NaOH aqueous solution was added until the pH reached to 9. The organic phase was washed with water, dried over anhydrous MgSO_4_, and concentrated under reduced pressure to yield yellow residue (1.0 g, 83.1%). ^1^H NMR (600 MHz, DMSO-*d_6_*) δ 8.92 (s, 1H), 8.83 (s, 1H), 8.58 (s, 1H), 8.32 (s, 1H), 7.62 (m, 1H), 7.44 (s, 1H), 7.31 (m, 1H), 7.14 (m, 3H), 7.08 (s, 1H), 6.85 (m, 1H), 3.93 (s, 3H), 3.83–3.87 (m, 4H), 3.65 (m, 1H), 2.37–2.41 (m, 2H), 2.05–2.08 (m, 2H), 1.92 (br, 1H). HRMS (m/z), [M + H]^+^ calculated for C_28_H_26_F_3_N_6_O_4_, 567.1968; found, 567.1948.

#### 4.1.19. *N-(4-((7-((1–(2-amino-2-oxoethyl)piperidin-4-yl)oxy)-6-methoxyquinazolin-4-yl)oxy)-3-*fluorophenyl*)-2–(2,6-difluorobenzylidene)hydrazine-1-carboxamide (18)*

To a suspension of **17** (1.0 g, 1.8 mmol) and Cs_2_CO_3_ (1.8 g, 5.4 mmol) in 10 ml DMF was added 2-chloroacetamide (0.33 g, 3.6 mmol). The resulting mixture was stirred for 8 h at 90 °C, and then poured into cold water. The precipitate was filtered off, washed with water, and dried to afford the title intermediate **18** as a dark yellow solid (0.68 g, 61.9%). HRMS (m/z), [M + H]^+^ calculated for C_30_H_29_F_3_N_7_O_5_, 624.2182; found, 624.2160.

#### 4.1.20. 2–(4-((4–(4-(3–(2-(2,6-Difluorophenyl)-4-oxotetrahydrothiophen-3-yl)ureido)-2-fluorophenoxy)-6-methoxyquinazolin-7-yl)oxy)piperidin-1-yl)acetamide (19)

To a suspension of **18** (0.31 g, 0.5 mmol) in dry CH_2_Cl_2_ (5 ml), mercaptoacetic acid (0.3 ml) and SiCl_4_ (15 drops) were added subsequently at 0 °C. The reaction mixture was allowed to cooled to room temperature, and refluxed for 6 h. The mixture was cooled to room temperature, and quenched by 2 ml cold water. After stirred for 5 min, 10% NaOH aqueous solution was added until pH reached to 10. The CH_2_Cl_2_ phase was separated and washed with water (2 × 5 ml), concentrated under reduced pressure to yield crude products which were purified by flash chromatography (eluent with 5–10% MeOH in DCM, 1% Et_3_N) to give target compound as a white solid (96.0 mg, 27.6%). ^1^H NMR (600 MHz, DMSO-*d_6_*) δ 9.21 (s, 1H), 8.90 (s, 1H), 8.53 (s, 1H), 7.57 (s, 1H), 7.55 (m, 1H), 7.48–7.53 (m, 2H), 7.35 (m, 1H), 7.21 (m, 1H), 7.17 (m, 2H), 6.16 (s, 1H), 4.77 (m, 1H), 3.98 (s, 3H), 3.81–3.85 (m, 2H), 3.07 (s, 2H), 2.73–2.94 (m, 4H), 2.10 (m, 2H), 1.84 (m, 2H). ^13 ^C NMR (101 MHz, DMSO-*d_6_*) δ 167.9, 163.5, 161.0, 159.3, 153.8, 153.2, 153.0, 150.3, 148.2, 137.6, 137.5, 133.2, 133.1, 131.0, 123.7, 114.1, 114.0, 111.8, 108.5, 108.0, 100.4, 73.5, 61.6, 56.2, 52.1, 51.1 (2 C), 30.7 (2 C), 29.7. HRMS (m/z), [M + H]^+^ calculated for C_33_H_32_F_3_N_6_O_6_S, 698.2008; found, 698.1991.

### 4.2. MTT assay

Taking lead compound BC2021-104511-15i, Fruquintinib and Regorafenib as positive controls, the cytotoxic activity against HT-29, HCT-116, COLO-205 and FHC cell lines by MTT assay. Detailed operation could be found in our previous study^29^.

### 4.3. Mobility shift assay

Kinase inhibitory activity against HGFR, MST1R, ABL, PDGFRβ, AXL, FLT3, RET, c-Src, and VEGFR-2 was evaluated by the mobility shift assay. Detailed operation could be found in our previous research^29^.

### 4.4. Molecular docking study

Docking study were conducted by Molecular Operating Environment 2018.01 (MOE, Chemical Computing Group ULC, Montreal, QC, Canada) using default settings. The structure of HGFR kinase was prepared (protonation, modelling of missing elements) from the original PDB files using Quickprepare. The binding site was defined within 5.0 Å of the cocrystallized ligands coordinates. The docking forcefield was Amber10: EHT. Ligand conformations were placed in the site with the Triangle Matcher method and ranked using the London dG scoring function.

### 4.5. Cell-Cycle analysis

COLO 205 cells (2.5 × 10^5^) were seeded in two 12-well plates and treated with DMSO and compound **10a** (0.11 μM, 0.33 μM and 1.0 μM) for 24 h (37 °C, 5% CO_2_). Cells were collected, centrifuged at 1000 rpm for 5 min, washed with cold PBS for twice, and then fixed with 500 μL 75% cold ethanol at 4 °C. The cells were washed with cold PBS and stained with propidium iodide for 30 min in the dark. Cell-cycle analyses were conducted with Cytoflex S (Beckman Coulter).

### 4.6. Annexin V-FITC/PI apoptosis assay

COLO 205 cells (2.5 × 10^5^) were seeded in two 12-well plates and treated with DMSO and compound **10a** (0.11 μM, 0.33 μM and 1.0 μM) for 72 h (37 °C, 5% CO_2_). The cells were collected, centrifuged at 1000 rpm for 5 min, and then washed with cold PBS for twice. Apoptosis assays were conducted with CytoFLEX S (Beckman Coulter).

## 5. Conclusions

Starting from the obtained HGFR and MST1R dual inhibitor BC2021-104511-15i, ten novel quinoline derivates were designed, synthesised and evaluated for their biological activity. More detailed SARs were summarised based on the kinase inhibitory activity and *in vitro* anticancer activity. Among these compounds, **10a** was identified as the most potent HGFR/MST1R dual inhibitor (HGFR IC_50_=0.11 μM and MST1R IC_50_=0.045 μM) with excellent anti-colorectal cancer activity (COLO 205 IC_50_=0.11 μM). Furthermore, it exhibited over 90-fold selectivity towards COLO 205 cells relative to human normal colorectal mucosa epithelial cell FHC cells. Docking study indicated that compound **10a** adopted an extended conformation as type II kinase inhibitor. H-bond, hydrophobic interaction and H-π interaction were the key contributors led to the strong binding affinity to kinase. Flow cytometry study demonstrated that compound **10a** could induce apoptosis in COLO 205 cells; however, it could not induce cell cycle arrest in COLO 205 cells. The results indicated that the anti-colorectal cancer activity against COLO 205 cells mainly depended on its cytotoxicity rather than antiproliferation. Preliminary kinase profile study showed that compound **10a** was a potential HGFR and MST1R dual inhibitor, its inhibitory activity against HGFR and MST1R was more potent than that of ABL, PDGFRβ, AXL, FLT3, RET, c-Src, and VEGFR-2 kinases.

## Supplementary Material

Supplemental Material
