# Supplementary material for: Discovery of novel conjugates of quinoline and thiazolidinone urea as potential anti-colorectal cancer agent
Source: J Enzyme Inhib Med Chem. 2022 Aug 31;37(1):2334–47. doi: 10.1080/14756366.2022.2117318 (PMC9448386; doi:10.1080/14756366.2022.2117318)
Supplement: Supplemental Material [file IENZ_A_2117318_SM7929.pdf]

Discovery of novel conjugates of quinoline and thiazolidinone urea as potential anti-colorectal cancer agent

Li Xiong[a,b], Huan He[a], Mengmeng Fan[a,b], Liping Hu[a,b], Fei Wang[a,b], Xiaomeng Song[a,b], Shengmin Shi[a,b], Baohui Qi[a,b,*]

[a]Department of Bioengineering, Zhuhai Campus of Zunyi Medical University, Zhuhai, China; [b]Key Laboratory of Biocatalysis & Chiral Drug Synthesis of Guizhou Province, Zunyi Medical University, Zunyi, China

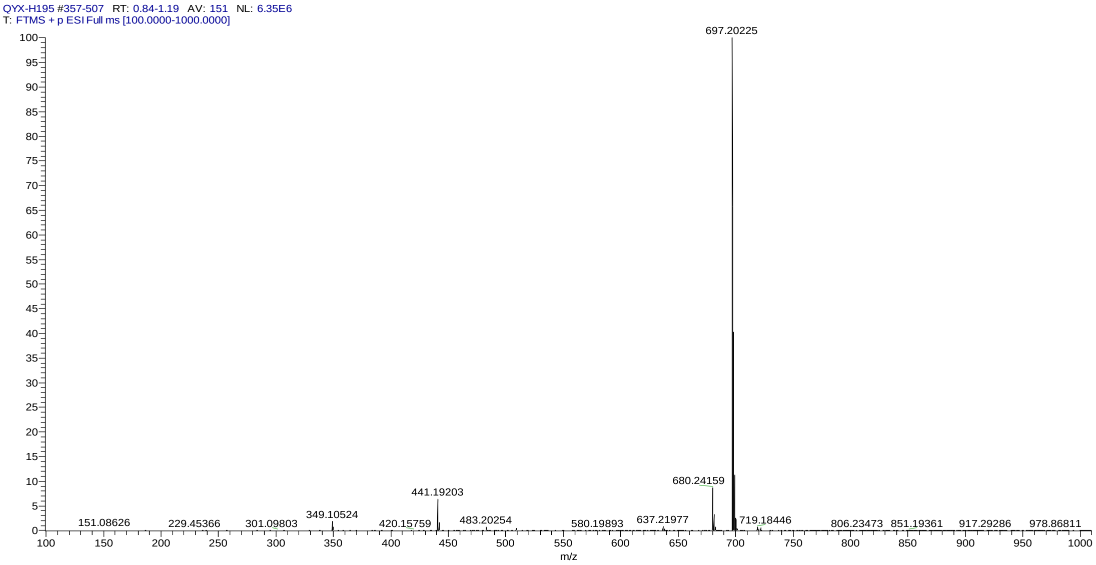

Fig. 1 HRMS of compound **10a**.

*Corresponding authors.
E-mail address: bhqi@zmu.gd.cn.

Information Classification: General

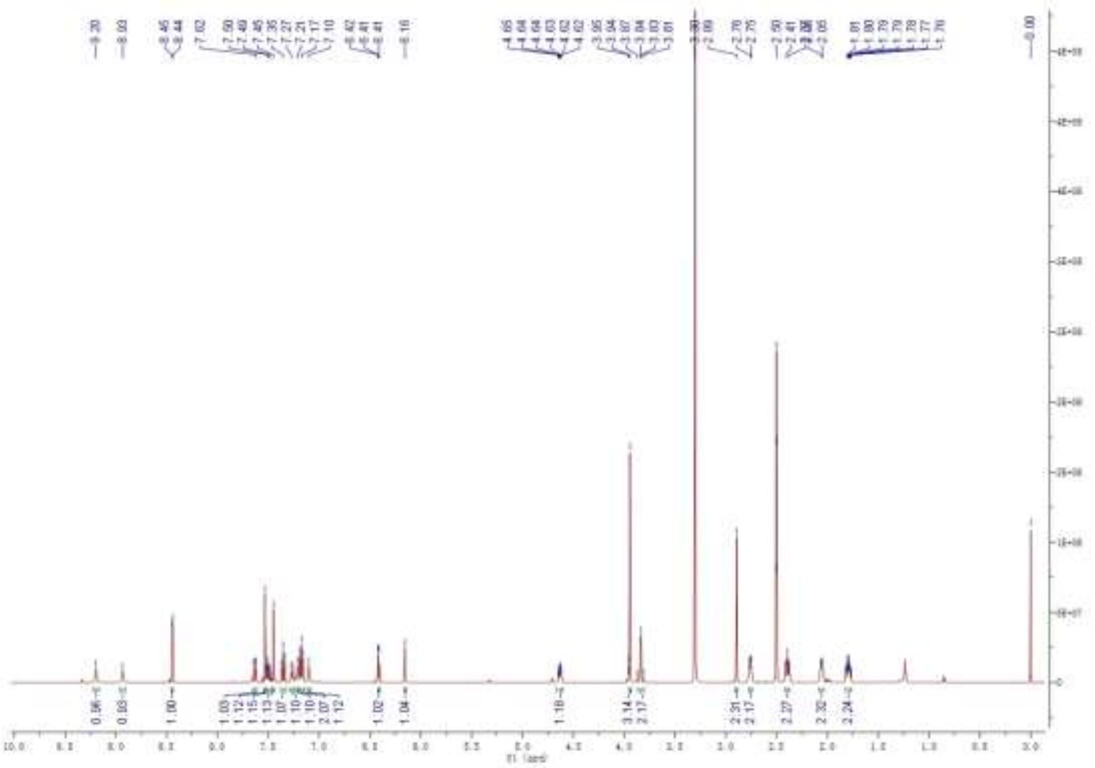

Fig. 2 ^{1}H NMR of compound **10a**.

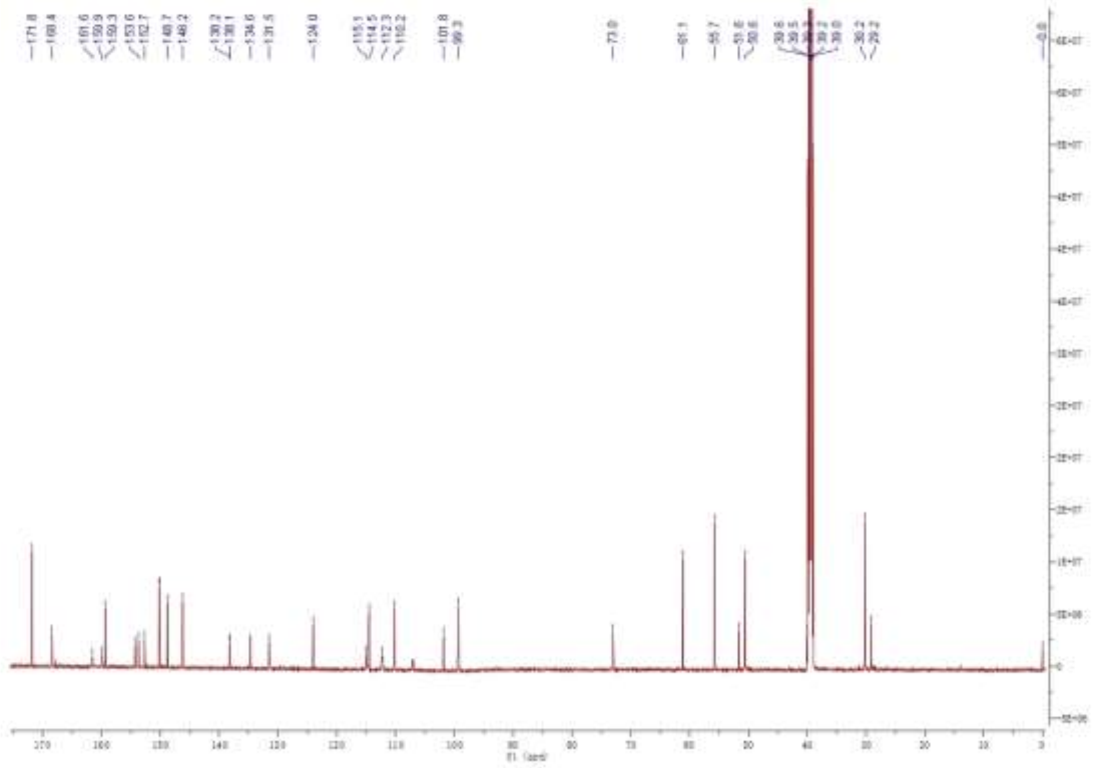

Fig. 3 ^{13}C NMR of compound **10a**.

Information Classification: General

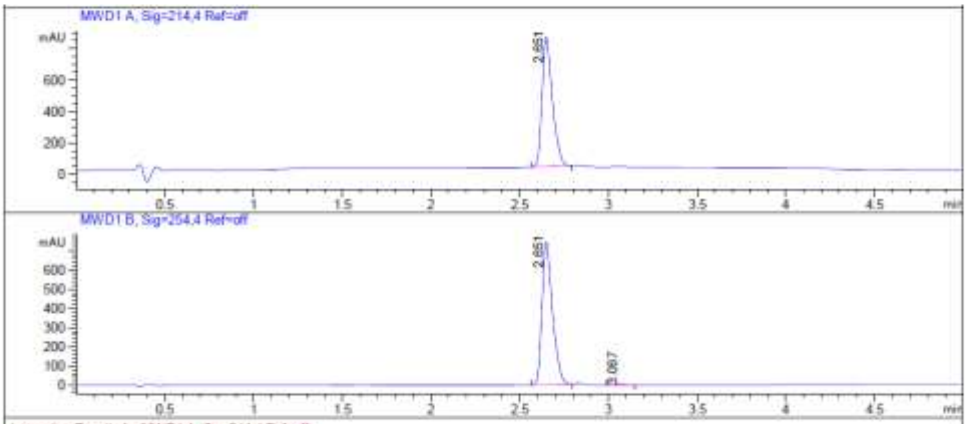

Integration Results for MWD1 A, Sig=214,4 Ref=off

RetTim	Width	Area	Height	Area%	MS(+)
2.65	0.06	3371.07	821.93	100.00	*** 67

Integration Results for MWD1 B, Sig=254,4 Ref=off

RetTim	Width	Area	Height	Area%	MS(+)
2.65	0.06	3861.16	745.78	99.75	*** 67
3.07	0.06	7.77	2.11	0.25	*** 10

Fig. 4 HPLC of compound **10a**.

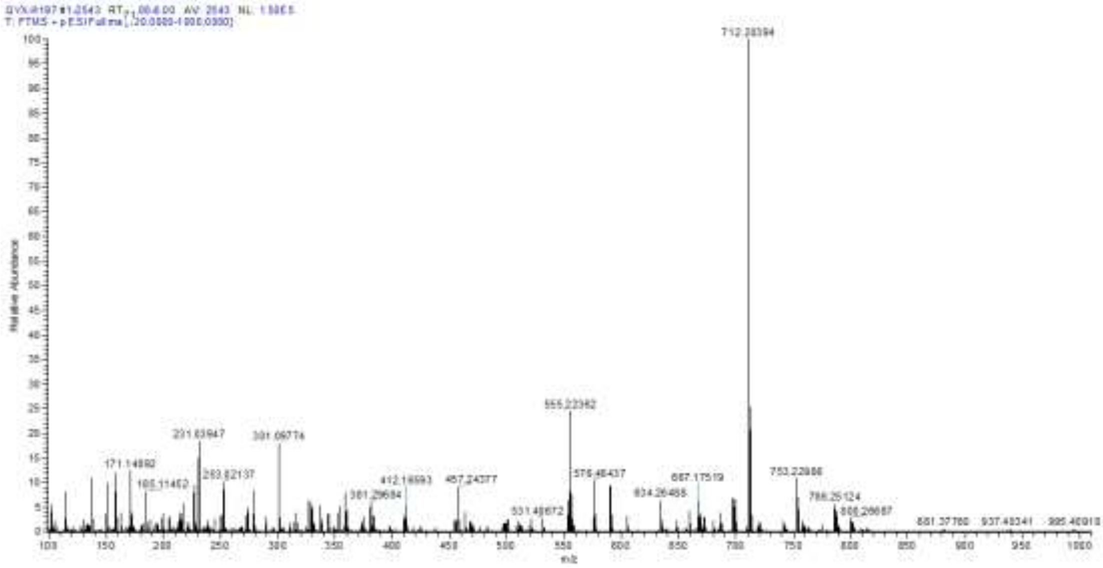

Fig. 5 HRMS of compound **10b**.

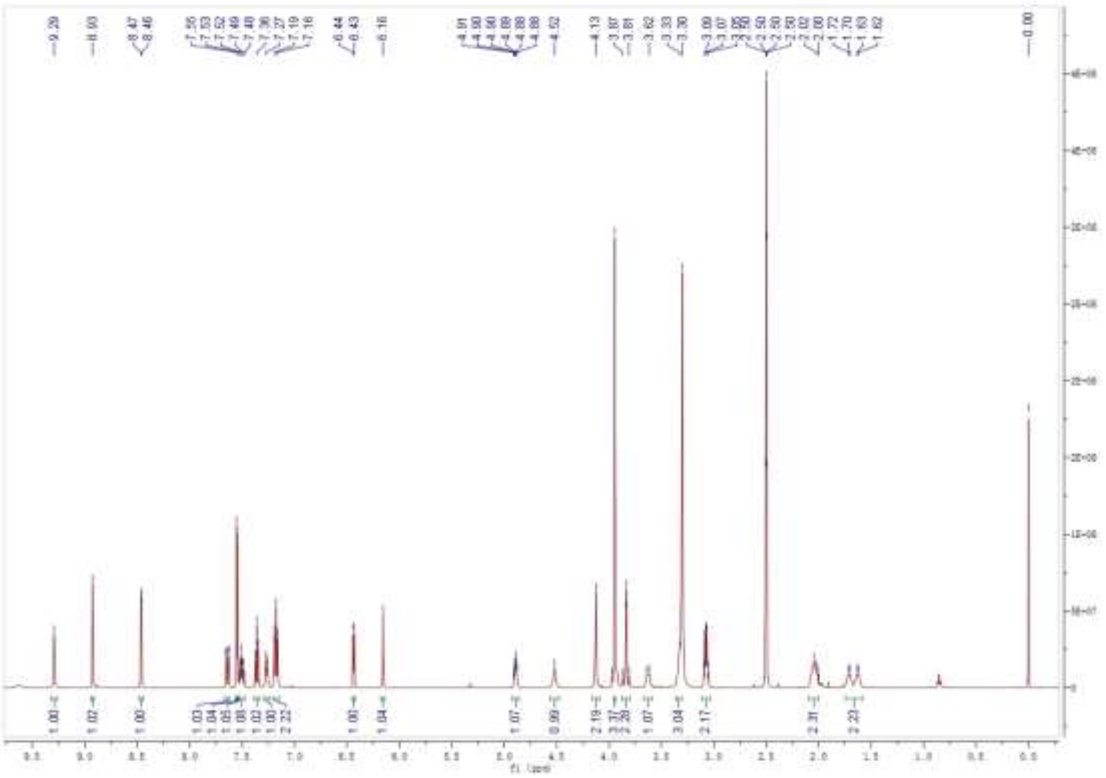

Fig. 6 ^{1}H NMR of compound **10b**.

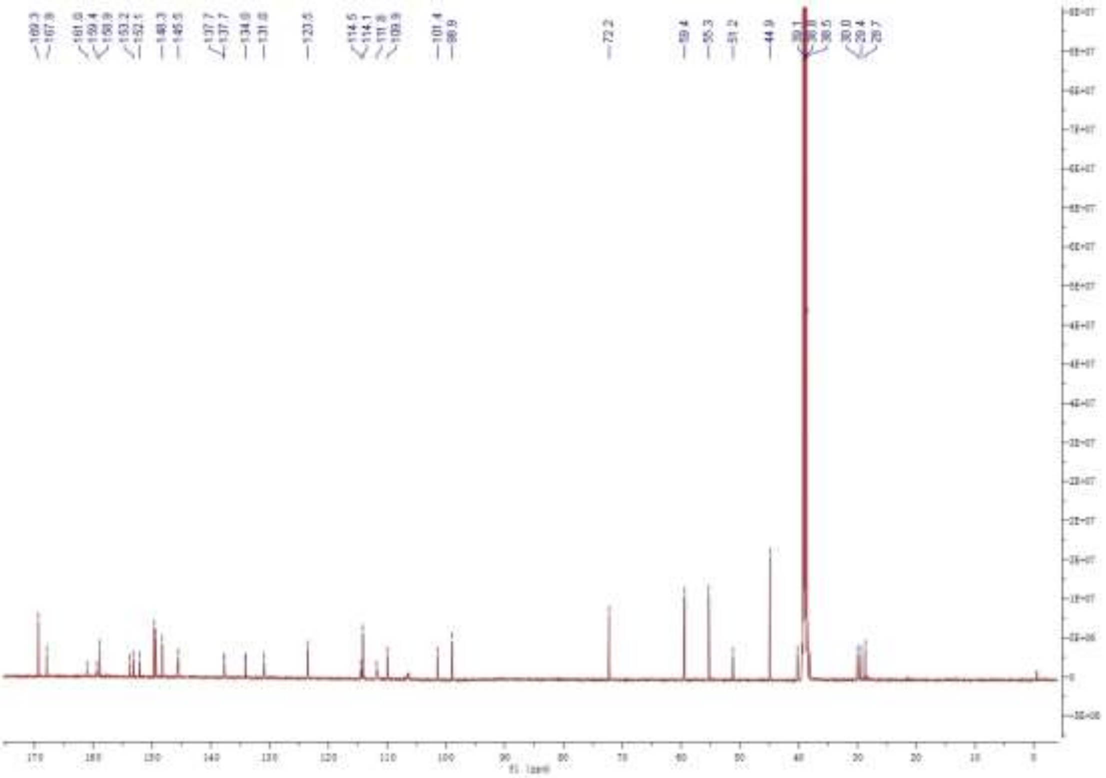

Fig. 7 ^{13}C NMR of compound **10b**.

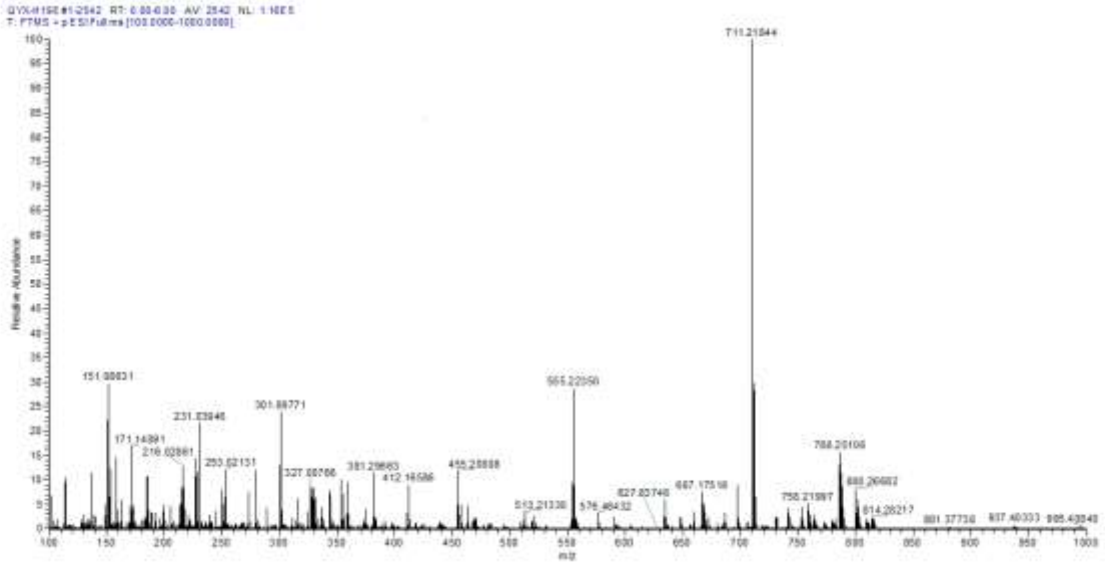

Fig. 8 HRMS of compound **10c**.

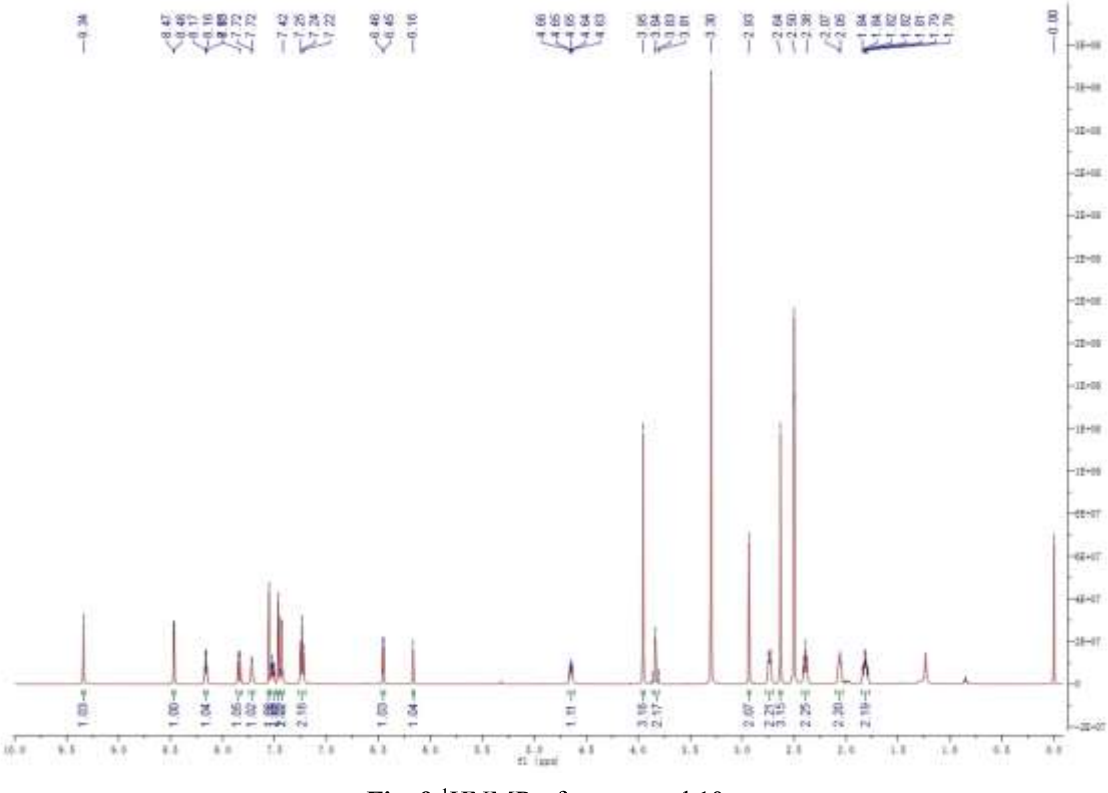

Fig. 9 ^{1}HNMR of compound **10c**.

Information Classification: General

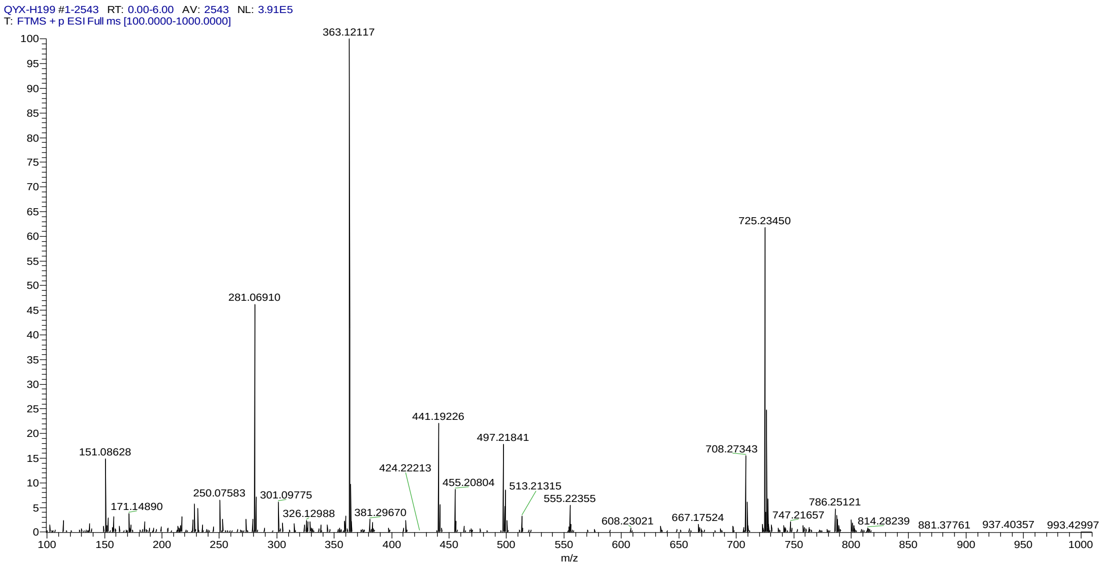

Fig. 10 HRMS of compound **10d**.

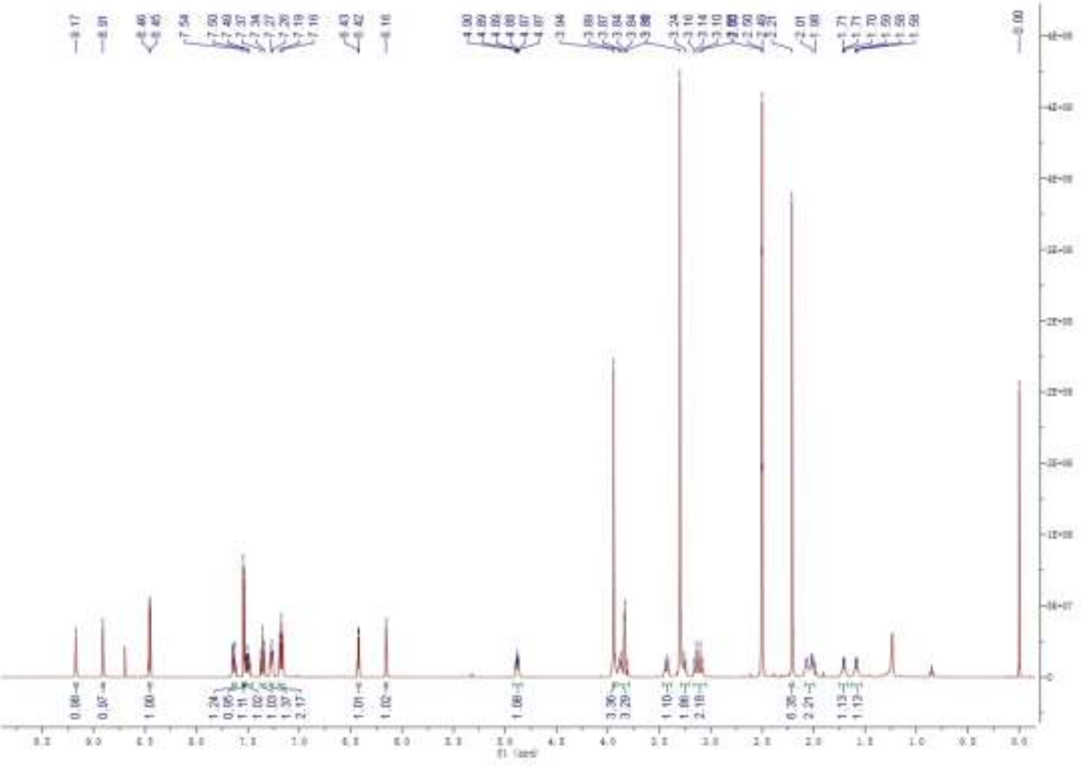

Fig. 11 ^{1}H NMR of compound **10d**.

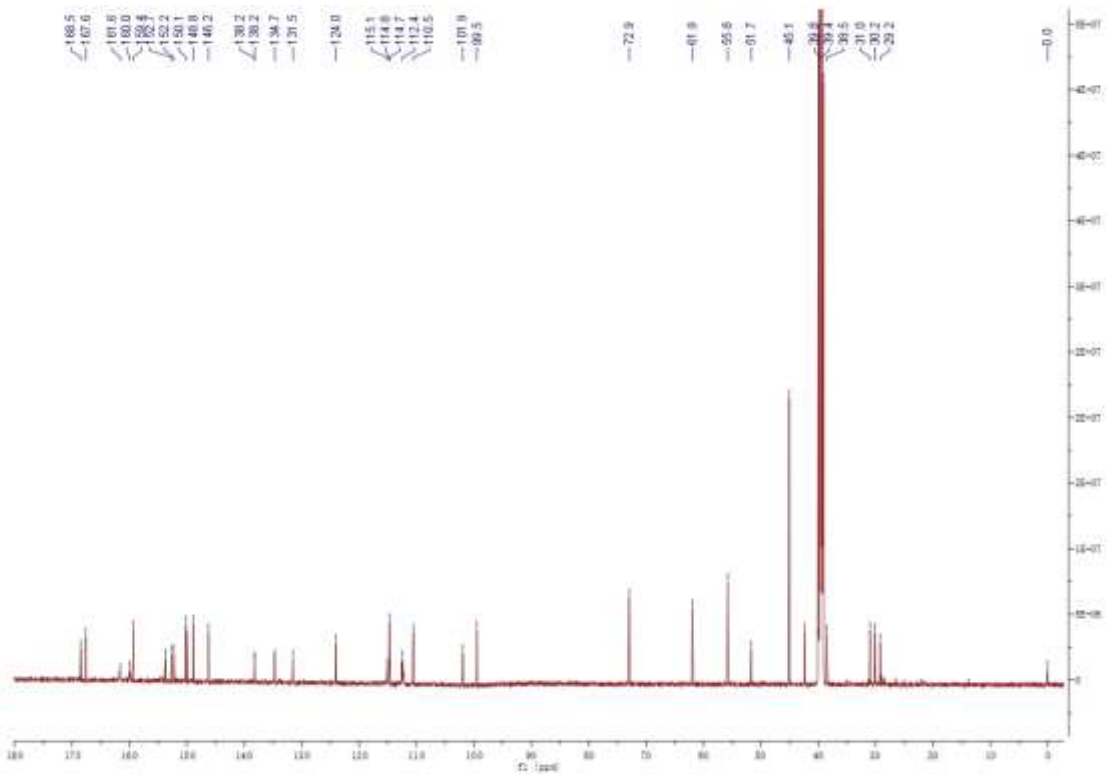

Fig. 12 ^{13}C NMR of compound **10d**.

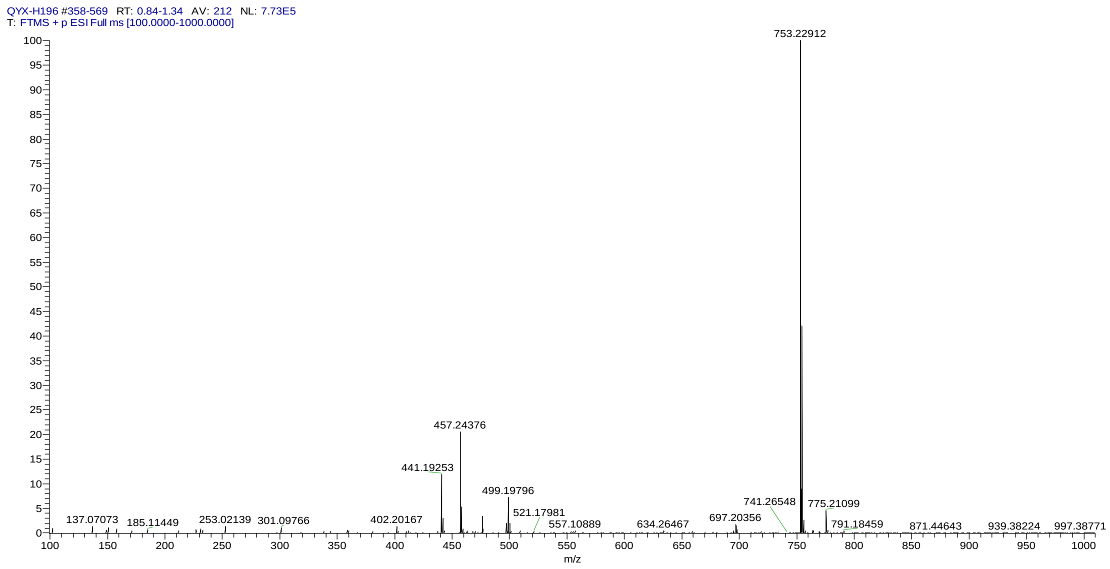

Fig. 13 HRMS of compound **10e**.

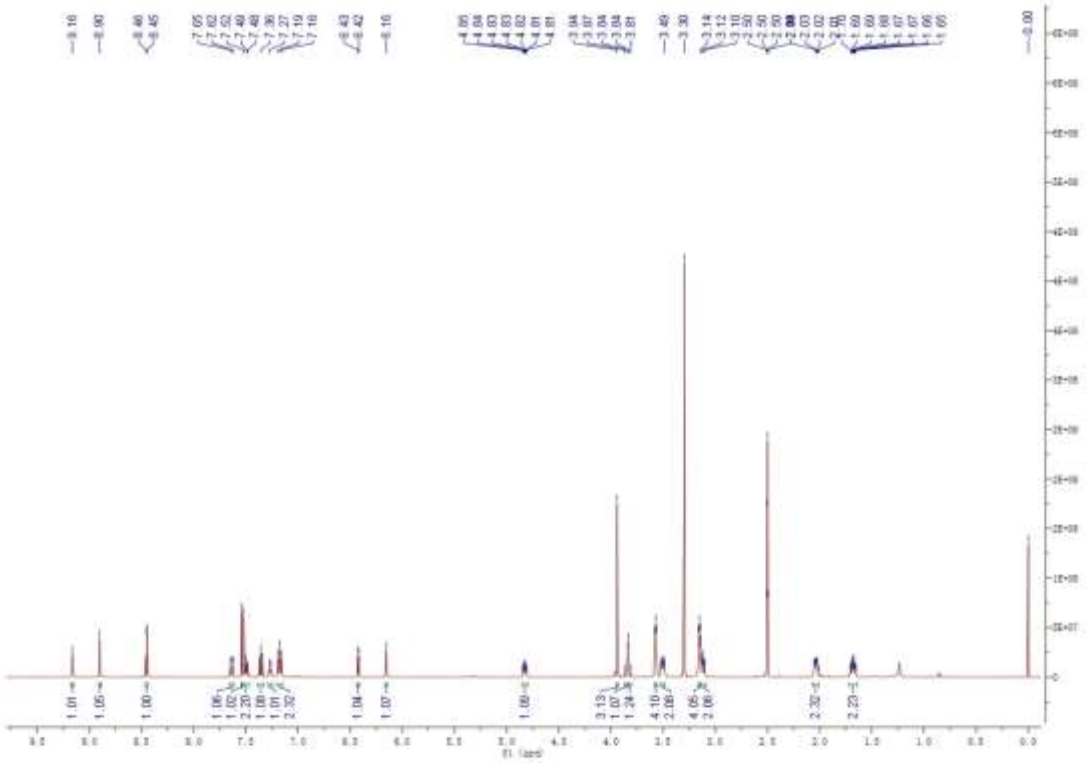

Fig. 14 ^{1}H NMR of compound **10e**.

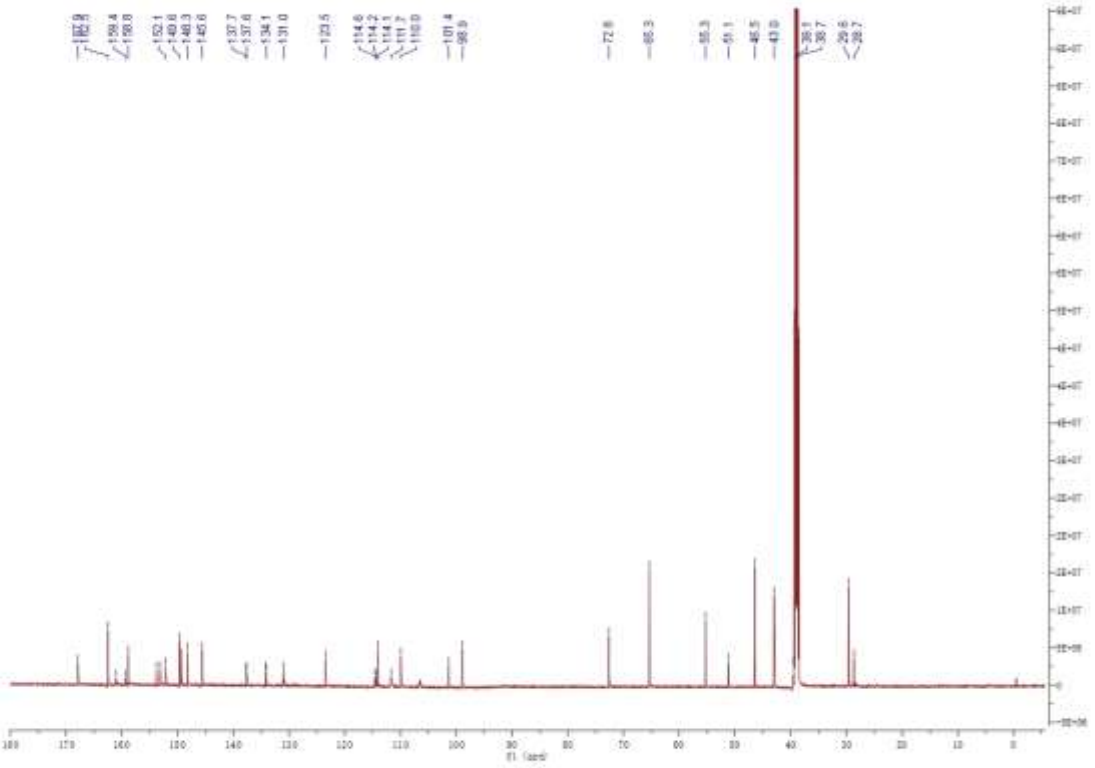

Fig. 15 ^{13}C NMR of compound **10e.**

Information Classification: General

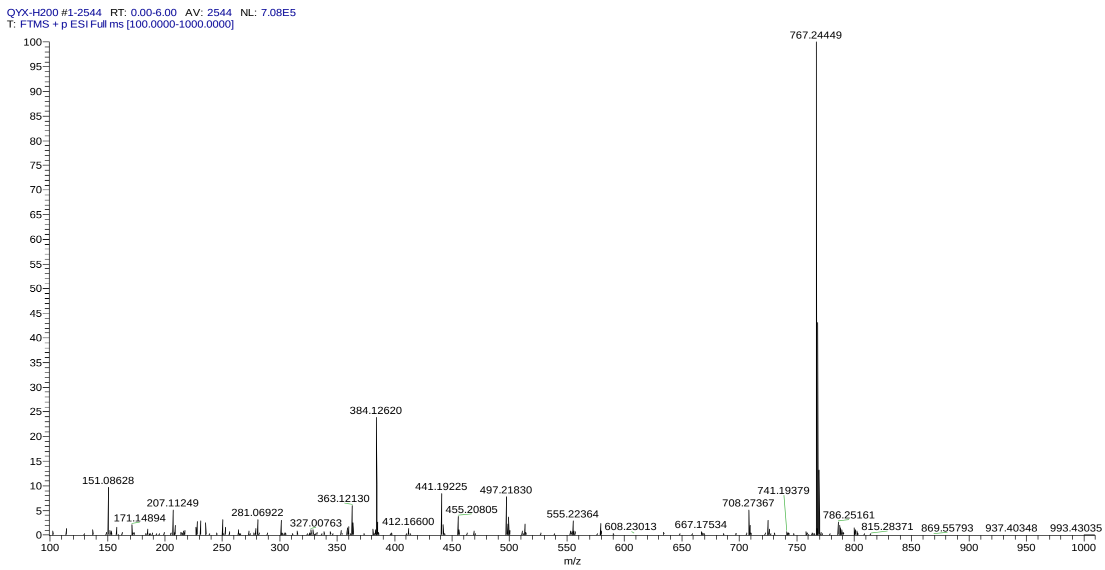

Fig. 16 HRMS of compound **10f**.

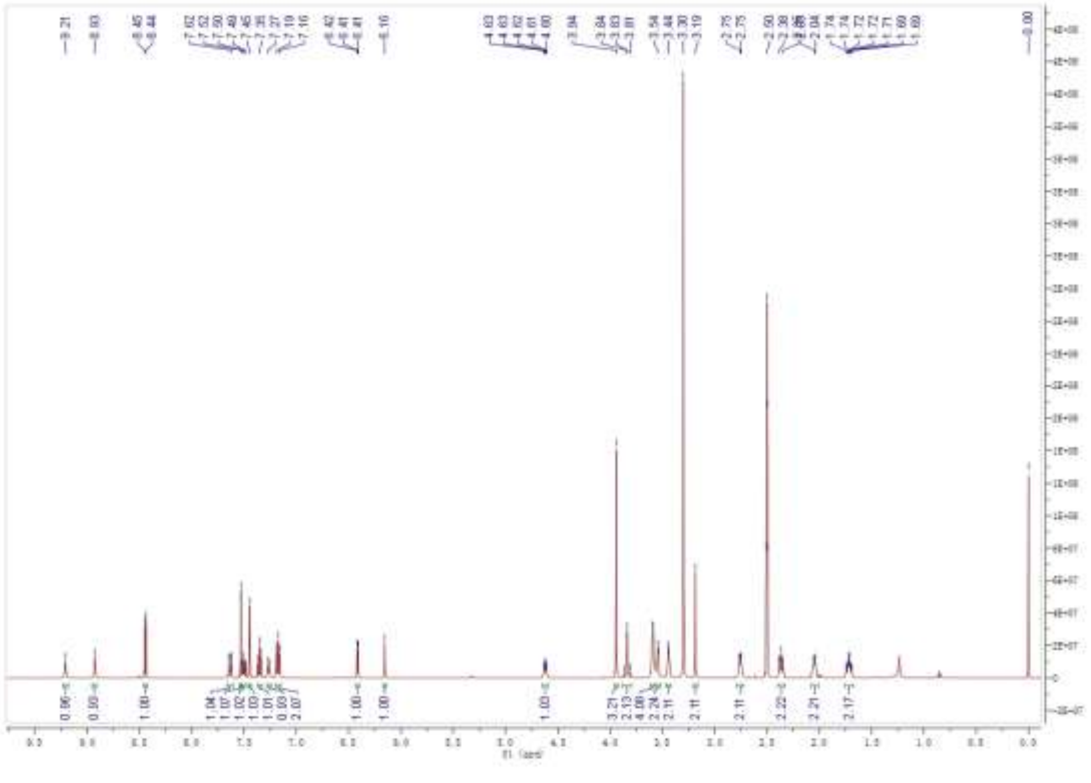

Fig. 17 ^{1}H NMR of compound **10f**.

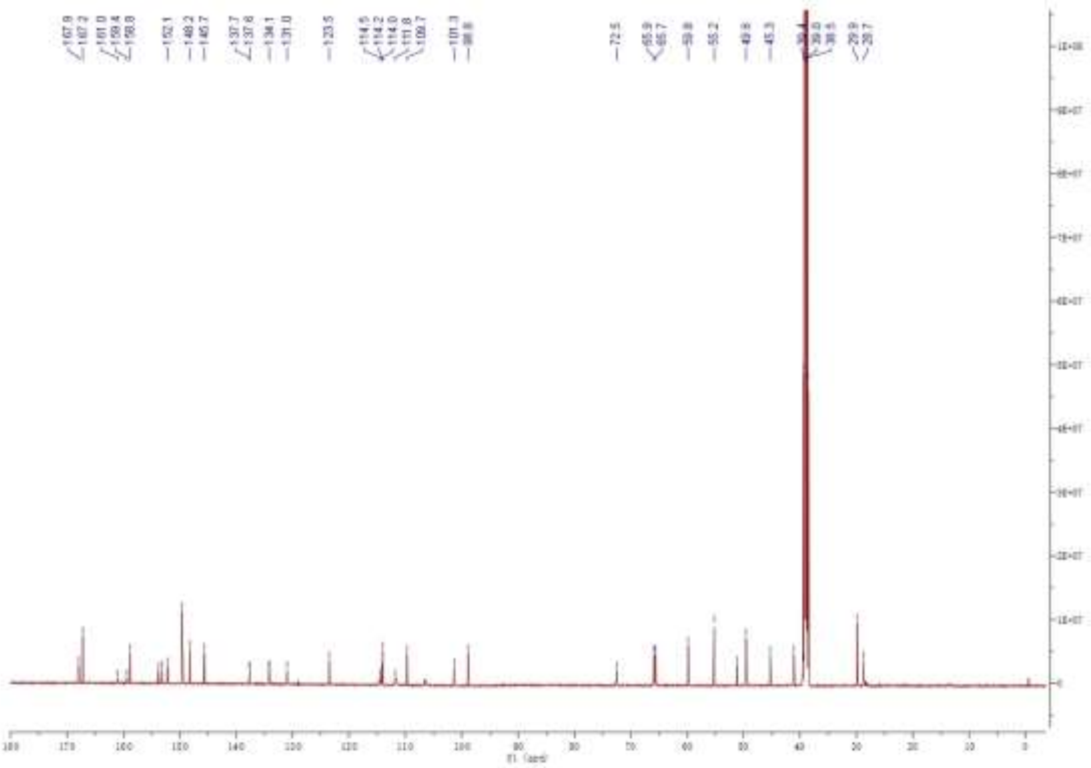

Fig. 18 ^{13}C NMR of compound **10f**.

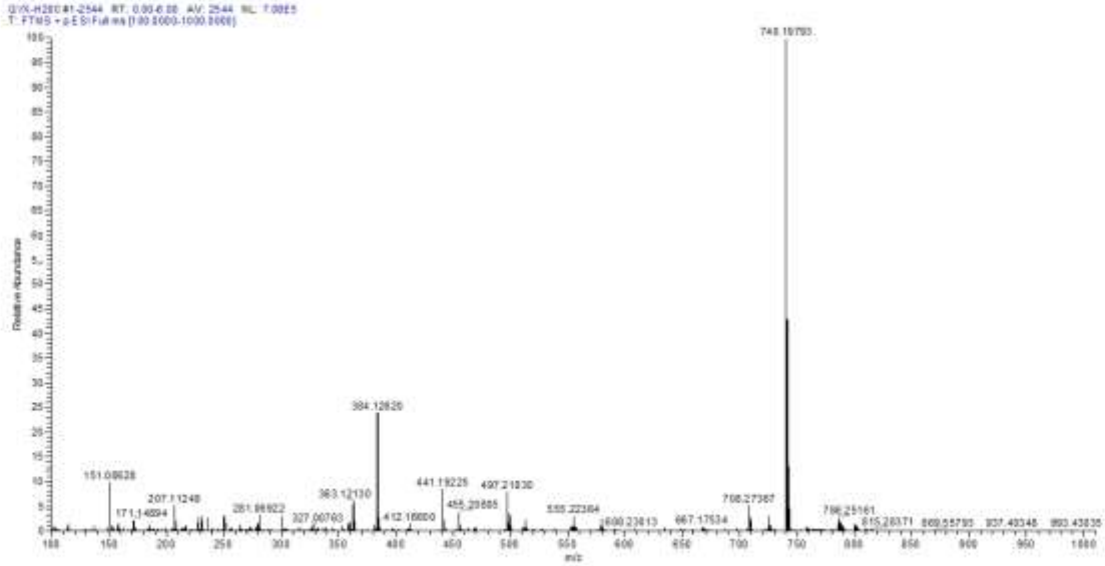

Fig. 19 HRMS of compound **10g**.

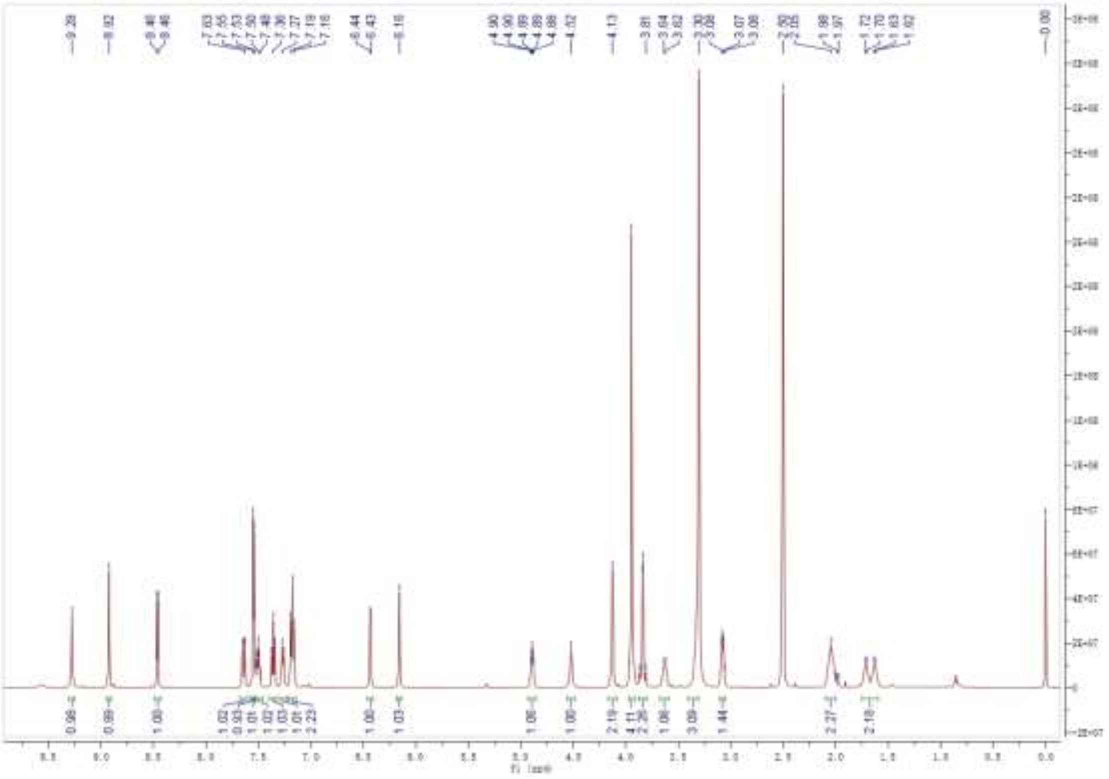

Fig. 20 ^{1}H NMR of compound **10g**.

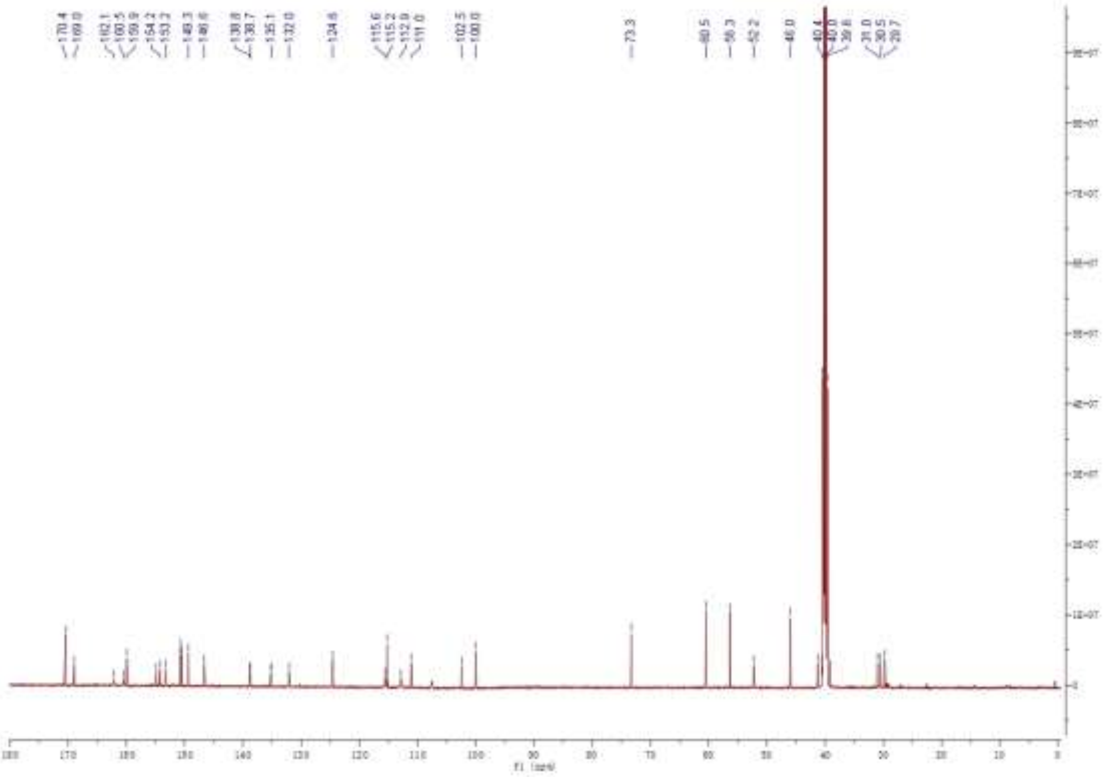

Fig. 21 ^{13}C NMR of compound **10g**.

Information Classification: General

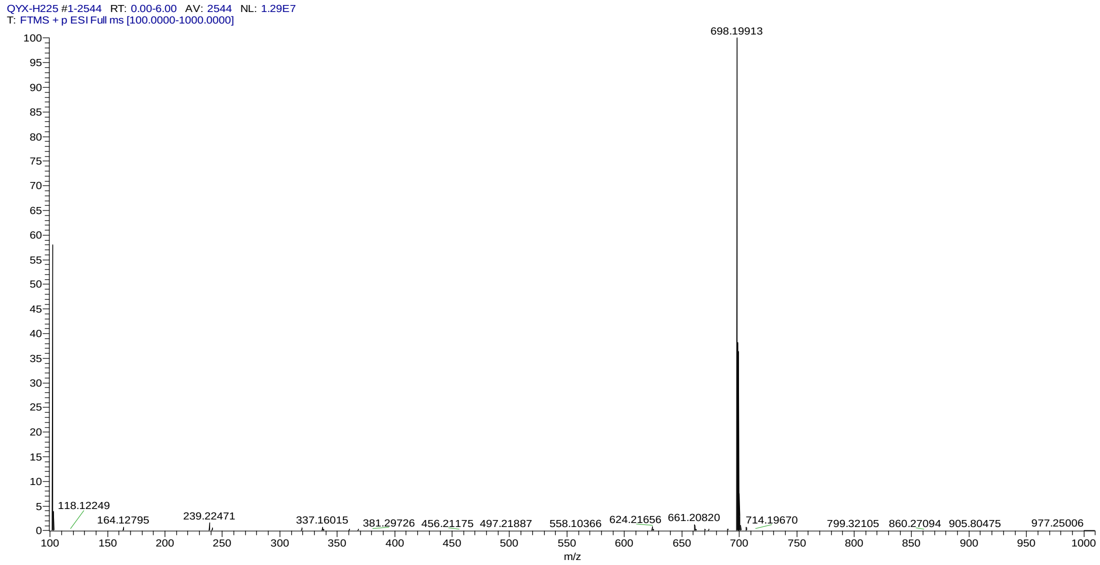

Fig. 22 HRMS of compound **19**.

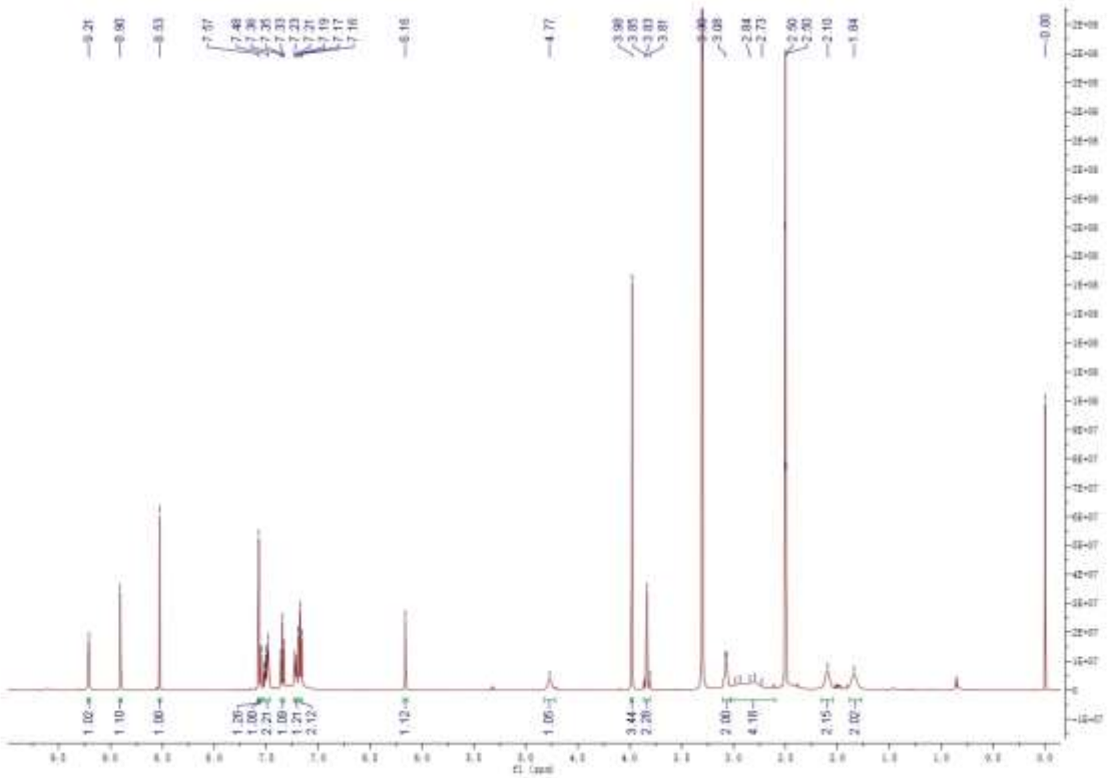

Fig. 23 ^{1}HNMR of compound **19**.

Information Classification: General

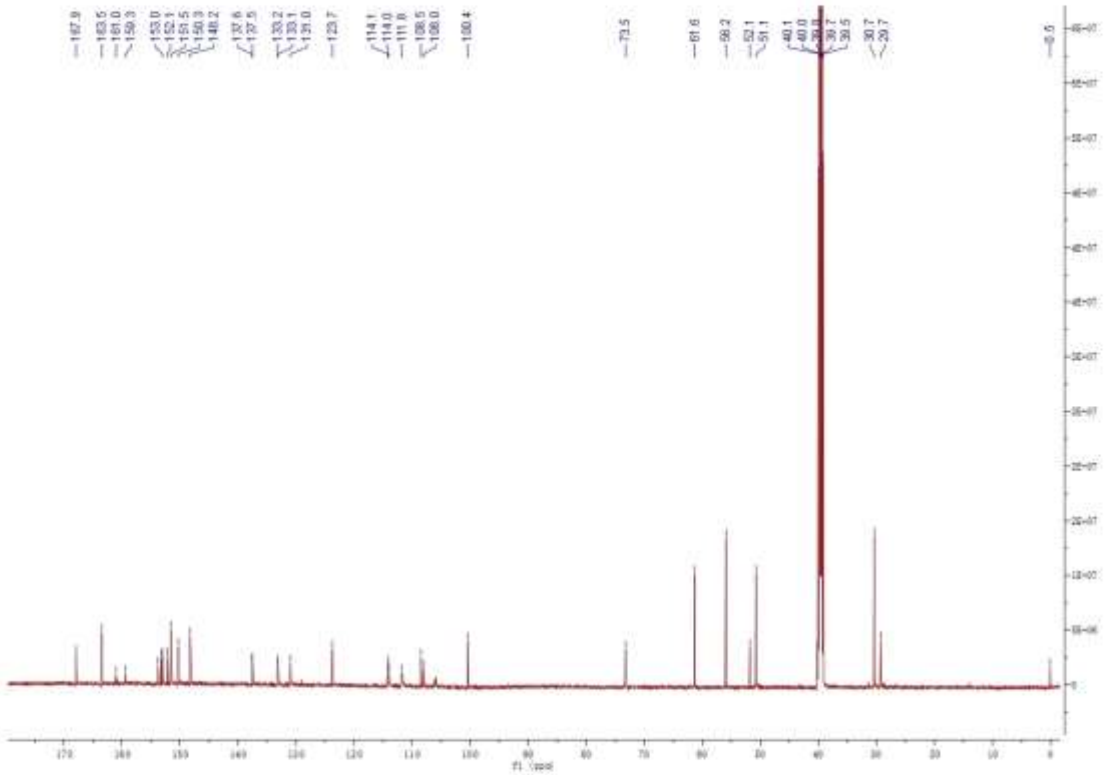

Fig. 24 ^{13}CNMR of compound **19**.

Information Classification: General
